# Lipid profiling of the filarial nematodes *Onchocerca volvulus, Onchocerca ochengi* and *Litomosoides sigmodontis* reveals the accumulation of nematode-specific ether phospholipids in the host

**DOI:** 10.1016/j.ijpara.2017.06.001

**Published:** 2017-12

**Authors:** Vera Wewer, Benjamin L. Makepeace, Vincent N. Tanya, Helga Peisker, Kenneth Pfarr, Achim Hoerauf, Peter Dörmann

**Affiliations:** a Institute of Medical Microbiology, Immunology and Parasitology, University Hospital Bonn, Sigmund-Freud-Str. 25, 53127 Bonn, Germany; b Institute of Infection and Global Health, University of Liverpool, Liverpool L3 5RF, UK; c Institut de Recherche Agricole pour le Développement, Regional Centre of Wakwa, BP65 Ngaoundéré, Cameroon; d Institute of Molecular Physiology and Biotechnology of Plants, University of Bonn, Karlrobert-Kreiten-Str. 13, 53115 Bonn, Germany; e German Center for Infection Research (DZIF), Partner Site Bonn-Cologne, Bonn, Germany.

**Keywords:** Filariasis, Biomarker, Diagnosis, Phospholipid, Human, Gerbil, Jird, Cattle

## Abstract

Onchocerciasis, a neglected tropical disease prevalent in western and central Africa, is a major health problem and has been targeted for elimination. The causative agent for this disease is the human parasite *Onchocerca volvulus*. *Onchocerca ochengi* and *Litomosoides sigmodontis*, infectious agents of cattle and rodents, respectively, serve as model organisms to study filarial nematode infections. Biomarkers to determine infection without the use of painful skin biopsies and microscopic identification of larval worms are needed and their discovery is facilitated by an improved knowledge of parasite-specific metabolites. In addition to proteins and nucleic acids, lipids may be suitable candidates for filarial biomarkers that are currently underexplored. To fill this gap, we present the phospholipid profile of the filarial nematodes *O. ochengi*, *O. volvulus* and *L. sigmodontis.* Direct infusion quadrupole time-of-flight (Q-TOF) mass spectrometry was employed to analyze the composition of phospholipids and their molecular species in the three nematode species. Analysis of the phospholipid profiles of plasma or serum of uninfected and infected hosts showed that nematode-specific phospholipids were below detection limits. However, several phospholipids, in particular ether lipids of phosphatidylethanolamine (PE), were abundant in *O. ochengi* worms and in bovine nodule fluid, suggesting that these phospholipids might be released from *O. ochengi* into the host, and could serve as potential biomarkers.

## Highlights

• Onchocerciasis is an infectious disease caused by filarial nematodes.
• Three different filarial nematodes infecting cattle, humans and jirds were studied.
• Phospholipids in nematodes and hosts were determined by mass spectrometry.
• Filaria-specific ether phosphatidylethanolamine (PE) lipids accumulate in the host.
• These ether PE lipids could serve as potential biomarkers for onchocerciasis.

## Graphical abstract

**Figure:**
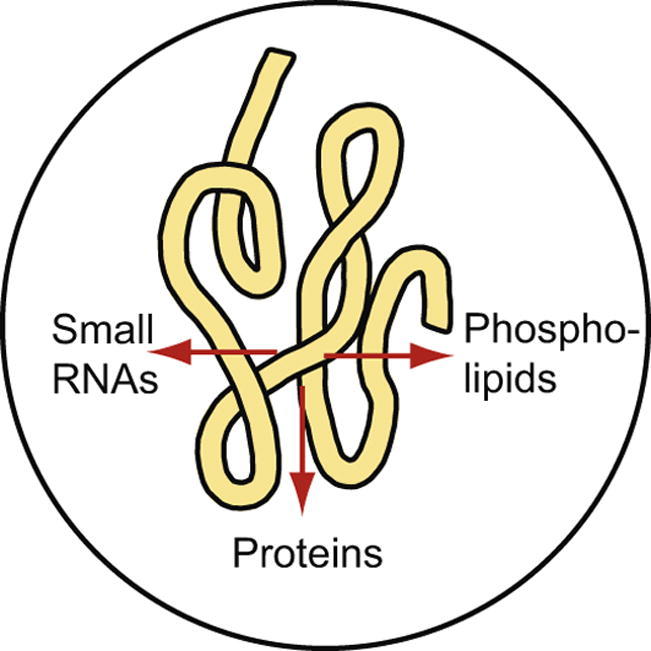

## 1 Introduction

Onchocerciasis, also known as river blindness, is a human parasitic disease prevalent in western and central Africa that is caused by infection with the filarial nematode *Onchocerca volvulus*. The nematodes are transmitted by *Simulium* spp., black flies. Female and male adult worms live in subcutaneous nodules, where they produce microfilariae (L1s) that enter the skin and can be ingested by black flies when they bite infected humans (Hoerauf et al., 2011). The adult worms are surrounded by nodule fluid which contains proteins and nucleic acids released by the parasites (Quintana et al., 2015, Armstrong et al., 2016). The most widely used treatment is administration of ivermectin, an effective microfilaricidal drug, which reduces the symptoms of the infection, such as blindness and skin disease, caused by the microfilariae. In addition, reduction of the microfilarial load contributes to preventing transmission. However, due to the longevity of the adult parasites, such therapies must be continued for several years until the adult worms reach the end of their reproductive lifespan (Allen et al., 2008).

In contrast to other filariae which may have blood-borne microfilariae (e.g., *Litomosoides sigmodontis*), the adult worms and microfilariae of *O. volvulus* are restricted to the subcutaneous tissues and skin, respectively. Therefore, the current diagnostic methods for determining *O. volvulus* infection are the detection of palpable nodules (onchocercomata) or the microscopic identification of microfilariae in skin snips. However, these methods are time consuming and require well trained individuals to recognize onchocercomata by palpation or to correctly identify the larvae (Hoerauf et al., 2011, Vlaminck et al., 2015). Additionally, as treatment with ivermectin becomes more widespread and successful, the current methods will not be useful for determining when to end ivermectin distribution to the communities, or for post-ivermectin monitoring. To facilitate screening of the population in endemic areas, a fast, inexpensive and non-invasive diagnostic test for onchocerciasis, i.e. live adult worms, would help to accelerate elimination programs. Previous diagnostic studies employed the measurement of antibodies in humans or the detection of filarial antigen in immuno (ELISA) assays (Chandrashekar et al., 1990, Cho-Ngwa et al., 2005). However, these immunoassays often lacked sensitivity and specificity. The currently available Ov-16 antibody test cannot distinguish between old and new infections, and even in children it can only indicate exposure to infection in young children (Vlaminck et al., 2015).

Research on biomarkers for onchocerciasis has focused on different target molecules including proteins, nucleic acids and metabolites. Recent studies have reported biomarkers for onchocerciasis, e.g., a modified metabolite of the nematode-derived neurotransmitter tyramine (N-acetyltyramine-O,β-glucuronide, NATOG), which was detected in human urine (Globisch et al., 2013), or nematode-specific small RNAs (Quintana et al., 2015), which were amplified from host plasma. While high sensitivity is critical for the detection of onchocerciasis biomarkers, specificity is equally important. In addition to onchocerciasis, a number of related diseases caused by infection with other filarial nematodes such as *Loa loa*, *Wuchereria bancrofti* or *Mansonella* spp. can occur in the same geographic area. Therefore, the identification of biomarkers with high specificity for *O. volvulus* is paramount. The required specificity might not be achieved employing a single biomarker. Therefore, a combination of different molecular classes might be crucial for the correct diagnosis and differentiation of the various filarial diseases.

Untargeted strategies to identify novel biomarkers usually rely on a global approach where a large number of different metabolites are extracted from the host material (e.g., plasma/serum or urine) (Denery et al., 2010, Globisch et al., 2013). Enrichment or purification of specific metabolite classes is often avoided, because this inevitably results in a loss of other metabolites. On the other hand, targeted analysis of specific metabolite classes (e.g., lipids) allows for higher sensitivity due to optimized extraction and purification. Furthermore, quantitative information can be obtained through the use of internal standards. To select the preferred metabolite class of the host to be included in such a targeted approach, it is important to first record metabolite patterns in both host and parasite, and reveal if any of the metabolites are abundant in, or even unique to, the parasite. It has been suggested that nematode-derived components could be released from the worm by excretion from the anus, active secretion from the secretory pore, as part of the “afterbirth” (uterine fluid etc.), wounding or turnover of the cuticle, natural death of the worm, or by partial cuticular shedding (Geary et al., 2012, Armstrong et al., 2014). Once such candidates have been identified in the worm, one can proceed to investigate their presence in infected compared with uninfected hosts.

In recent years, MS-based lipidomic approaches have been increasingly employed in the search for lipid biomarkers as diagnostic tools for different diseases. Lipids are the building blocks of membranes which form the barriers between the cells. They are involved in signal transduction and nutrient exchange, and establish the contact sites during host-pathogen interactions. Alterations in lipid profiles are used for the diagnosis of hereditary disorders of lipid metabolism including Gaucher disease and acquired disorders such as diabetes (Hu et al., 2009, Shui et al., 2011), or prostate cancer (Tung et al., 2008, Hu et al., 2009, Zhou et al., 2012). Lipids were the focus of a number of studies on *O. volvulus* and related filarial nematodes in the past (Maloney and Semprevivo, 1991, Smith et al., 1996, Mpagi et al., 2000, Wuhrer et al., 2000, Denery et al., 2010), however, most lipid studies were conducted before advances in LC-MS techniques that allow the high throughput analysis of minute amounts of sample material. In addition, due to limitations of sample material, only a few studies focused on lipid analysis of *O. volvulus*. Therefore most studies used material from related nematode species. For example, the most comprehensive study on non-polar lipids and phospholipids in *Onchocerca* spp. was performed using a parasite of cattle, *Onchocerca gibsoni* (Maloney and Semprevivo, 1991), with thin-layer chromatography (TLC). The phospholipid classes each comprise a large number of molecular species, with each molecular species harboring two fatty acyl moieties attached to the glycerol backbone. Methods such as TLC cannot provide information about molecular species composition, which could serve as putative biomarkers. In other reports, TLC and gas chromatography (GC) were combined to analyze the lipid classes and fatty acids of *Brugia malayi* (Smith et al., 1996, Longworth et al., 1987) and of the avian parasite *Ascaridia galli* (Ghosh et al., 2010). However, no targeted analysis of phospholipid molecular species of *O. volvulus* and related nematodes by state-of-the-art LC-MS technology has, to our knowledge, been conducted to date.

Research on onchocerciasis in humans is restricted by ethical issues, but can be facilitated using appropriate animal infections. Several such animal models have been introduced into filariasis research (Allen et al., 2008). *Litomosoides sigmodontis* infection of the Mongolian gerbil (“jird”, *Meriones unguiculatus*) represents a rodent model especially suited for laboratory research and studies on immunology. The veterinary infection of cattle with *Onchocerca ochengi* is of particular interest due to the very close phylogenetic relationship to the human parasite *O. volvulus*, and therefore it can serve as a model for *O. volvulus* infections (Morales-Hojas et al., 2007). Similar to onchocerciasis in humans, *O. ochengi* microfilariae are transmitted by *Simulium* spp., and the adult worms produce collagenous nodules that, in contrast to *O. volvulus*, are intradermal (Makepeace and Tanya, 2016). In addition to genetics and disease progression, metabolic similarities and differences between the various related organisms are of interest as well.

In the present study, we compared the phospholipid profiles of three different filarial nematodes, *O. ochengi*, *O. volvulus* and *L. sigmodontis,* employing direct infusion nano-electrospray ionization (ESI) quadrupole time-of-flight (Q-TOF) mass spectrometry. In the search for putative biomarkers, phospholipids were also analyzed in blood plasma or serum of uninfected humans, cattle and jirds, or in hosts infected with *O. volvulus, O. ochengi*, or *L. sigmodontis,* respectively. In the course of these experiments, we identified nematode-specific molecular species of phospholipids in nodule fluids, which therefore represent potential biomarkers for filarial diseases. This information will be valuable for developing novel diagnostic tools and for future metabolomics studies using one of the three parasitic infections.

## 2 Materials and methods

### 2.1 *Onchocerca ochengi*, bovine serum and nodule fluid

Adult *O. ochengi* worms and nodule fluid were obtained from the Ngaoundéré cattle abattoir, Adamawa Region, Cameroon, as previously described (Quintana et al., 2015, Armstrong et al., 2016). Worms were shipped frozen in PBS, thawed, and washed with water before lipid extraction to remove the buffer. In total, four samples each of male worms and of non-gravid female worms were used for lipid analysis. Bovine serum was sampled from naturally infected adult animals and uninfected calves obtained from the Adamawa Region, Cameroon, during the European Union Seventh Framework Programme Research grant (contract 131242) “Enhanced Protective Immunity Against Filariasis (EPIAF)”, (http://cordis.europa.eu/project/rcn/94066_en.html) and the “Rapid and high-throughput diagnosis of *Onchocerca volvulus* infections“ (RADIO) project (funded by the Bill & Melinda Gates Foundation). All procedures performed on animals in Cameroon were equivalent to those authorized by a Home Office Project License (Animals (Scientific Procedures) Act 1986) for related work on cattle in the UK. The study was also approved by a local Animal Welfare Ethics Committee commissioned by the Cameroon Academy of Sciences. The 15 animals used for the supply of onchocerciasis-positive plasma had a median nodule load of 62.

### 2.2 Onchocerca volvulus worm material and human plasma

Human plasma samples from patients infected with *O. volvulus* were collected and archived as part of the EPIAF EU project (see Section 2.1). Ethical clearance for the study and the use of archived samples for biomarker research was obtained from the Committee on Human Research Publication and Ethics at the University of Science and Technology in Kumasi, Ghana, and the Ethics Committee at the University of Bonn, Germany (Arndts et al., 2014). In compliance with the Helsinki Declaration on the use of humans in biomedical research, study participants received detailed information on the nature and objectives of the study and those who agreed to take part in the study signed an informed consent form. Participation was voluntary. Infected individuals were identified by palpation of onchocercomata and identification of microfilariae by microscopy of skin biopsies as described (Arndts et al., 2014). Three pooled *O. volvulus* samples obtained after collagenase treatment of excised nodules containing (i) two males, (ii) three females and two males, and (iii) three females and one male, were used for lipid measurements.

### 2.3 Litomosoides sigmodontis worms and *M. unguiculatus* plasma

Animal plasma and adult *L. sigmodontis* worms were obtained from Mongolian gerbils (“jirds”, *M unguiculatus*) housed in accordance with the European Union animal welfare guidelines using protocols approved by the Landesamt für Natur, Umwelt und Verbraucherschutz, Cologne, Germany (AZ 8.87-51.05.30.10.038) (Al-Qaoud et al., 1997, Globisch et al., 2015). Four samples each of female and of male *L. sigmodontis* worms and plasma from uninfected and infected jirds were used for lipid analysis. All samples were frozen in liquid nitrogen and stored at −80 °C.

### 2.4 Lipid extraction

The high sensitivity of the Q-TOF mass spectrometer equipped with a nanospray-ESI source allowed the measurement of lipids in extremely small volumes of sample material. For female *O. ochengi* worms, approximately 100 mg (wet weight) of sample were used for lipid extraction. Plasma or serum (100 µl) was used for phospholipid analysis from bovine and human samples. For the analysis of bovine nodule fluid, 5–25 µl were used. For male *L. sigmodontis*, where only very small amounts of worm material were available, no wet weight values were recorded. The sample volume was reduced to 50 µl for jird plasma due to limited availability.

Lipid extraction from worms was performed as described (Bligh and Dyer, 1959). Worm tissue was homogenized in the presence of chloroform/methanol (1:2), cell debris was pelleted by centrifugation, and the supernatant containing lipids was harvested. The pellet was re-extracted at least three times with chloroform/methanol (2:1). The combined supernatants were purified by a phase-partitioning step with water. The lower organic phase containing lipids was harvested and dried.

Lipids were extracted from 50–100 µl of plasma by addition of three volumes of chloroform/methanol (2:1), two volumes of 1 M KCl and three volumes of chloroform. The mixture was vigorously shaken and phase separation was achieved by centrifugation for 15 min at 1,000*g*. The lower organic phase was harvested and the upper phase re-extracted with three volumes of chloroform. The two organic phases were combined and dried. The dried lipids were dissolved in 10 volumes of chloroform and stored at −20 °C.

### 2.5 Direct infusion nanospray ESI Q-TOF MS/MS

Phospholipids were analyzed by direct infusion nanospray ESI Q-TOF MS/MS on an Agilent 6530 Q-TOF mass spectrometer with nano-ESI chipcube technology. Q-TOF MS parameters were set as described previously (Wewer et al., 2011). Lipids were dissolved in methanol/chloroform/300 mM ammonium acetate (665:300:35, v/v/v) for analysis (Welti et al., 2002). Phospholipids were quantified in relation to internal standards by Q-TOF MS/MS analysis as described (Welti et al., 2002, Gasulla et al., 2013). Standards for phosphatidylcholine (PC), phosphatidylethanolamine (PE) or phosphatidylinositol (PI) were purchased from Avanti Polar Lipids (Alabaster, USA) or Matreya (State College, USA). The ions used for quantification are listed in Supplementary Tables S2–S7.

## 3 Results

### 3.1 Quantification of phospholipids in *O. ochengi*, *O. volvulus* and *L. sigmodont*

**Fig. 1:**
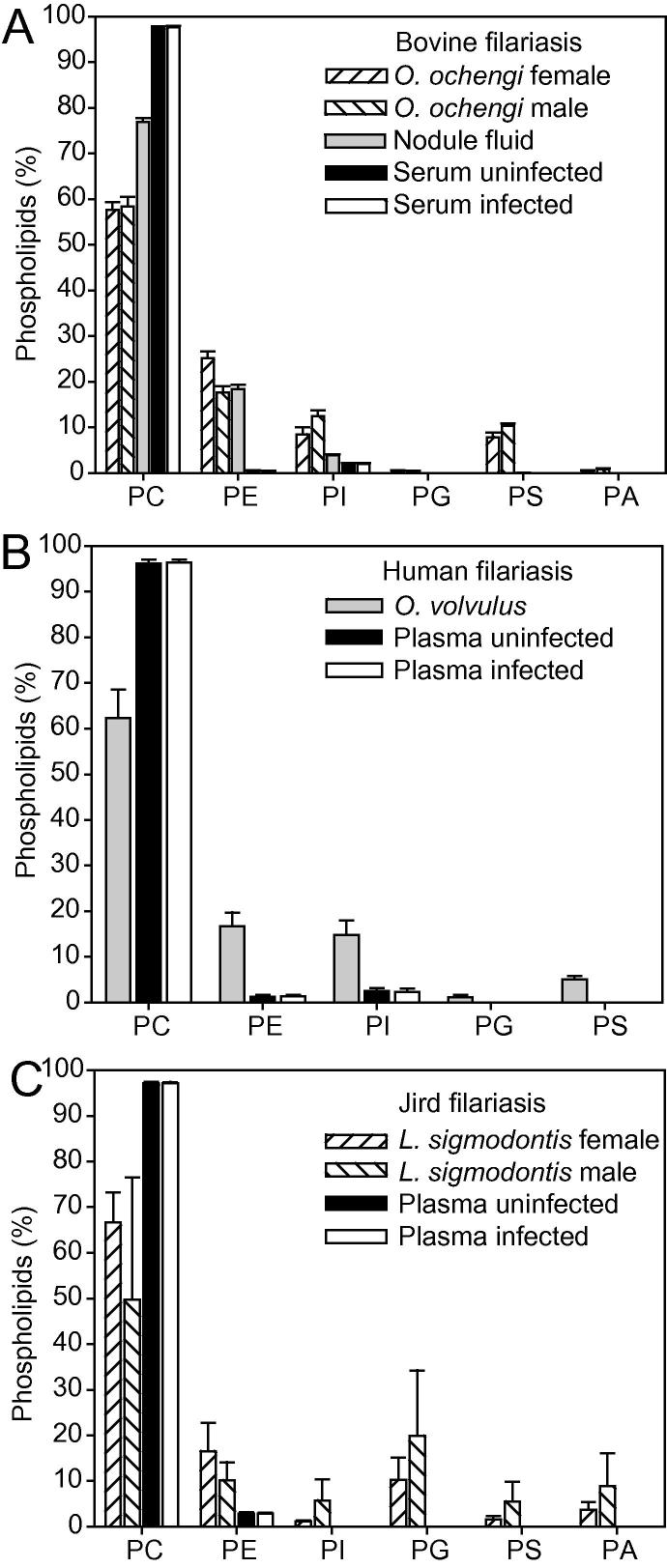
Quantification of phospholipids in nematodes, nodule fluid and plasma or serum of bovine, human and jird filarial infections. (A) Phospholipids extracted from *Onchocerca ochengi*, nodule fluid or from serum of uninfected or infected cattle. (B) Phospholipids extracted from *Onchocerca volvulus* or from plasma of uninfected or infected patients. The *O. volvulus* values represent the mean ± S.D. of three different worm samples (one containing only male worms, and two containing both sexes, but biased towards females; individual values are shown in Supplementary Table S1). (C) Phospholipids extracted from *Litomosoides sigmodontis* and plasma from uninfected or infected jirds. Phospholipids were measured by quadrupole time-of-flight (Q-TOF) mass spectrometry. Mean ± S.D. (*n* = 4). PA, phosphatidic acid; PC, phosphatidylcholine; PE, phosphatidylethanolamine; PG, phosphatidylglycerol; PI, phosphatidylinositol; PS, phosphatidylserine.

*Onchocerca ochengi* is the closest known relative to *O. volvulus* (Morales-Hojas et al., 2007), and therefore is used as a model to study onchocerciasis. In contrast to humans, *O. ochengi* worms can be isolated in larger numbers from cattle and employed for lipid analysis. Lipid extracts from male and female *O. ochengi* were isolated and measured by direct infusion MS to identify sex-dependent components, differences between bovine nodule fluid and serum, and for comparison with the phospholipid patterns of *O. volvulus* and *L. sigmodontis*. Female *O. ochengi* contained 57.6% PC followed by 25.2% PE, 8.5% PI and 7.8% phosphatidylserine (PS) (Fig. 1A). The phospholipid distribution in male *O. ochengi* worms was similar, with a reduced proportion of PE (17.6%), accompanied by increased amounts of PI (12.4%) and PS (10.4%). Phosphatidylglycerol (PG) and phosphatidic acid (PA) levels were very low in female and male *O. ochengi* worms, amounting to less than 1% of total phospholipids.

*Onchocerca volvulus* worm material can only be obtained from subdermal nodule preparations from infected patients. Due to highly limited access, a mixture of male and female *O. volvulus* nematodes was analyzed and averages were calculated and plotted (Fig. 1B; Supplementary Table S1). One sample contained only male worms, the other two were biased towards females. PC represents the major phospholipid in *O. volvulus* with 62.4%, followed by PE with 16.7% and PI with 14.8% (Fig. 1B). Lower amounts of PS (5.0%) and PG (1.1%) were detected. The phospholipid composition of the individual specimens suggested that females contain lower amounts of PC (∼58%, ♀; ∼69%, ♂), but higher levels of PE (∼18%, ♀; ∼14%, ♂) and PG (∼17%, ♀; ∼11%, ♂) (Supplementary Table S1).

While *O. ochengi* is more closely related to *O. volvulus*, *L. sigmodontis* is important for pharmacological studies because the complete life cycle can be maintained in mice or jirds (Allen et al., 2008). We analyzed phospholipids in *L. sigmodontis* to compare the results with *O. ochengi* and *O. volvulus*. Analogous to *O. ochengi*, the phospholipids from females and males were recorded separately for comparison (Fig.1C). PC was the most abundant phospholipid in *L. sigmodontis*, followed by PE and PG, while PI, PS and PA were minor components. The amounts of PC and PE in females were higher than in males, while the levels of the other phospholipids (PI, PG, PS, PA) were slightly lower.

Taken together, PC was by far the most abundant phospholipid in the three filarial nematodes with concentrations of ∼60%. PE was the next most abundant phospholipid, while the other phospholipids (PI, PG, PS, PA) were lower and differed between the three nematodes. The phospholipid compositions of females and males were similar, with some minor differences, e.g., higher levels of PE in females from all three nematodes.

### 3.2 Filarial nematodes contain high amounts of ether lipids of PC and PE

**Fig. 2:**
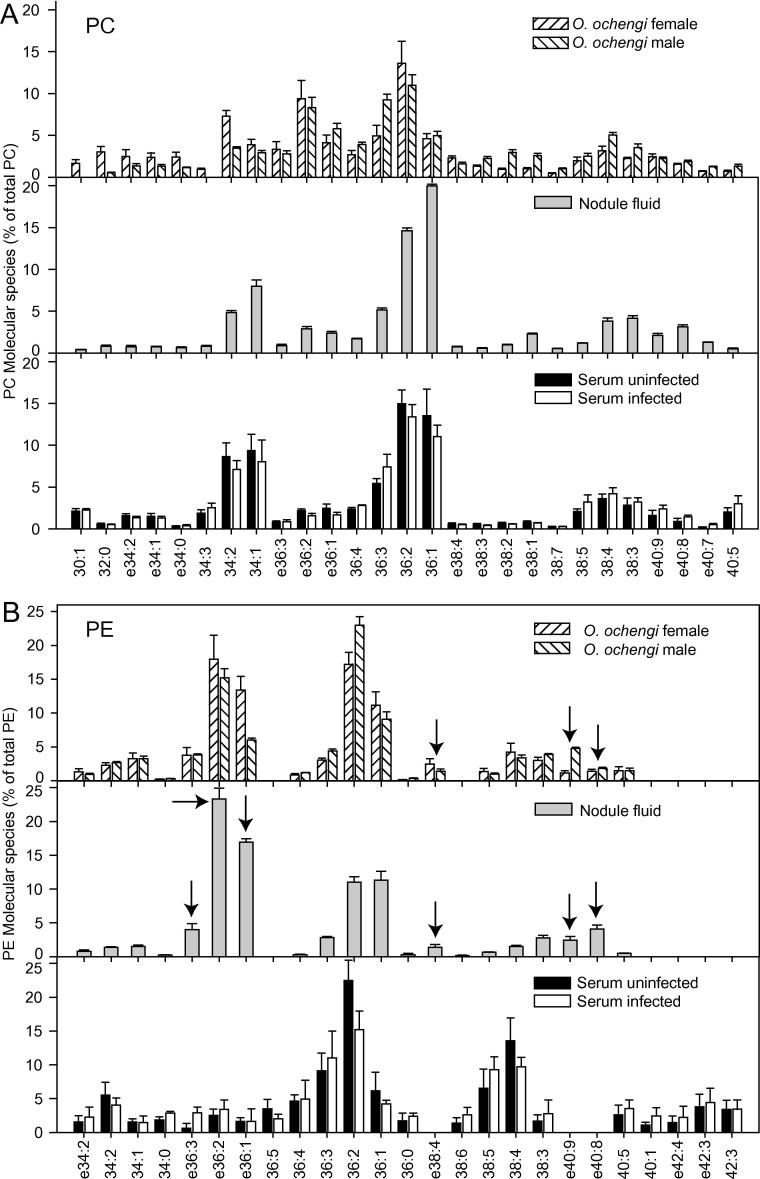
Phospholipid molecular species distribution in *Onchocerca ochengi,* nodule fluid and bovine serum. (A) Phosphatidylcholine (PC). (B) Phosphatidylethanolamine (PE). Only molecular species representing at least 2% (calculated on mol basis) of total PE or PC in one of the sample sets are shown. Mean ± S.D. (*n* = 4). Arrows indicate ether lipids of PE specific for *O. ochengi*.

**Supplementary Fig. S1:**
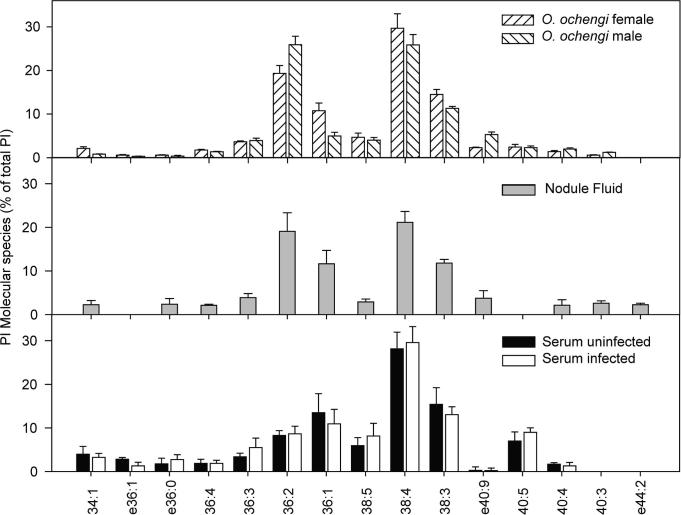
Phospholipid molecular species distribution of phosphatidylinositol (PI) in *Onchocerca ochengi,* nodule fluid and bovine serum. Only molecular species that represented at least 2% (calculated on mol basis) of total PI in one of the sample sets are shown. Means ± S.D. (*n* = 4). ePI, ether-PI.

Q-TOF mass spectrometry analysis not only provides the absolute amounts of phospholipid classes, but in addition generates data for the molecular species composition of each phospholipid. Fig. 2A and B and Supplementary Fig. S1 show the molecular species composition for PC, PE and PI, respectively, of female and male *O. ochengi* worms. PC and PE contain considerable amounts of 34:2, 36:1, 36:2, 36:3, 38:3 and 38:4 molecular species (total number of carbon atoms:total number of double bonds in the acyl chains) carrying C16, C18 or C20 fatty acids. Interestingly, ether lipids of PC and PE carrying a long chain alcohol bound in ether linkage to the glycerol instead of a fatty acid were highly abundant in *O. ochengi*. The ether lipids (e)36:2 PC and e36:1 PC amounted to 9.4% and 4.1% in female *O. ochengi* worms. The ether PE level in female *O. ochengi* was even higher, as e36:2 PE and e36:1 PE represented 18.0% and 13.4% of total PE, respectively. Further ether lipids detected in *O. ochengi* were e38:4, e40:9 and e40:8 molecular species of PC and PE. In contrast to PC and PE, PI from *O. ochengi* was not enriched with ether lipids. Instead, 36:2 PI, 38:3 and 38:4 PI were the most abundant molecular species (Supplementary Fig. S1). The ether lipids e36:2 PI and e36:1 PI were below detection limits, while e40:9 PI was of low abundance.

**Fig. 3:**
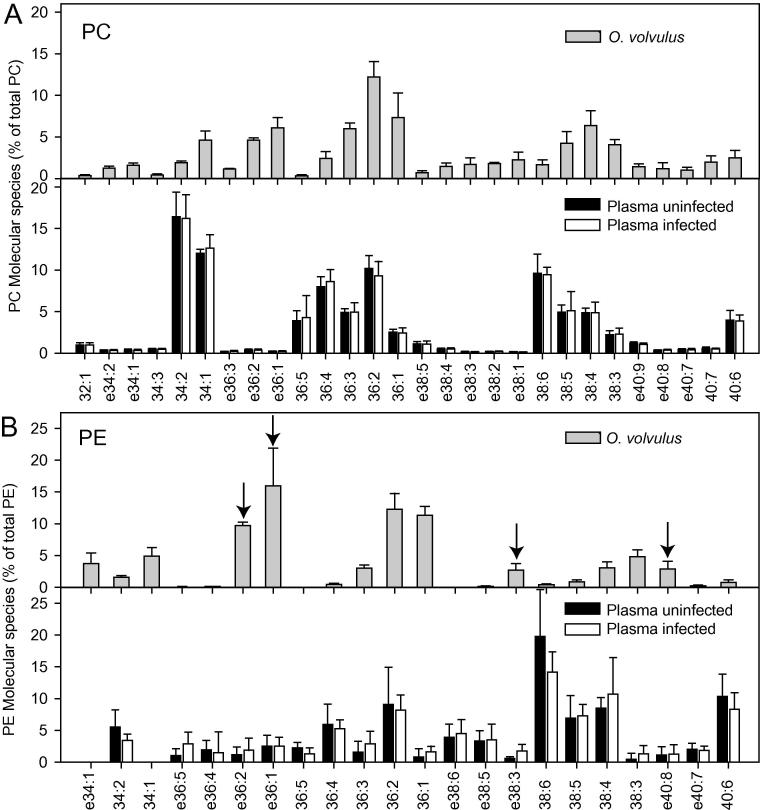
Phospholipid molecular species distribution in *Onchocerca volvulus* worms and human plasma. (A) Phosphatidylcholine (PC). (B) Phosphatidylethanolamine (PE). Only molecular species representing at least 2% (calculated on mol basis) of total PE or PC in the samples are shown. The *O. volvulus* values represent the mean ± S.D. of three different worm samples (one containing only male worms and two containing both sexes but biased towards females). The plasma samples represent means ± S.D. (*n* = 4). ePC, ether-PC; ePE, ether-PE (plasmalogen). Phospholipid molecular species are abbreviated according to the total number of carbon atoms:combined number of double bonds in the two acyl moieties. Arrows indicate ether lipids of PE specific for *O. volvulus*.

**Fig. 4:**
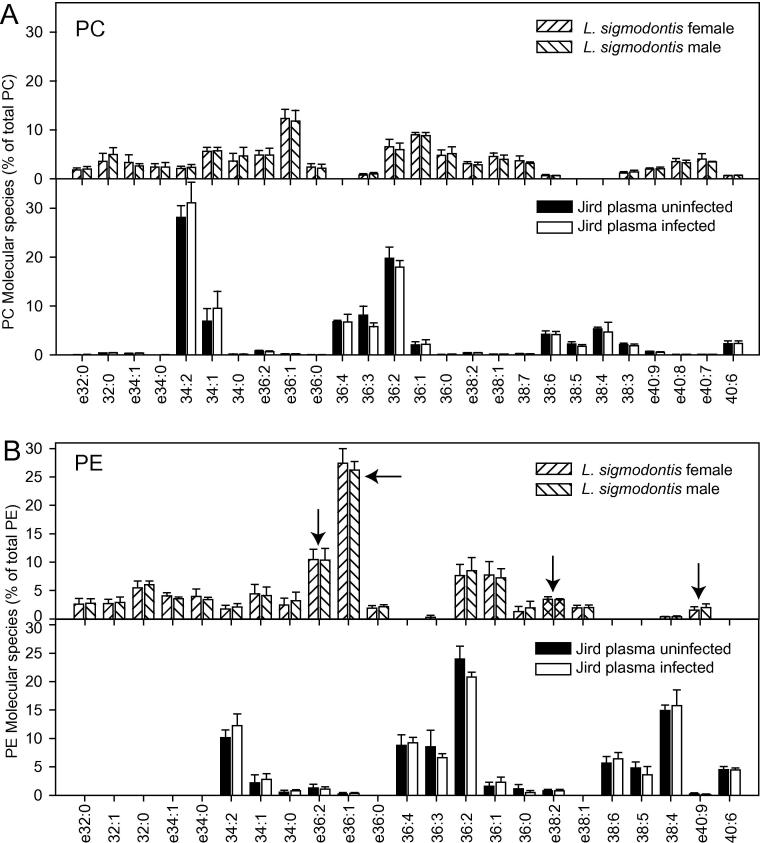
Phospholipid molecular species distribution in *Litomosoides sigmodontis* and jird plasma. (A) Phosphatidylcholine (PC). (B) Phosphatidylethanolamine (PE). Only molecular species that represented at least 2% (calculated on mol basis) of total PE or PC in one of the sample sets are shown. Mean ± S.D. (*n* = 4). Arrows indicate ether lipids of PE specific for *L. sigmodontis*.

Similar to *O. ochengi*, molecular species of 34:1, 36:1, 36:2, 36:3, 38:3, 38:4, 38:5 and the ether lipids e36:2, e36:1, e40:9 and e40:8 of PC and PE were abundant in *O. volvulus* worms (Fig. 3A and B). Together, ether lipids represented more than 10% of total PC or PE in *O. volvulus*. Furthermore, the phospholipid composition of *L. sigmodontis* was recorded and compared with *O. ochengi* and *O. volvulus*. The most abundant PC and PE molecular species in *L. sigmodontis* were 32:0, 34:1, 36:1 and 36:2 and, and the ether lipids e36:1 PC with 12.4% of total PC and e36:1 PE with 27.4% of total PE (Fig. 4). Taken together, the molecular species composition of PC and PE were similar in all three filarial nematodes. In addition to the “normal” acyl-linked molecular species, PC and PE of the worms contained high amounts of ether phospholipids; in particular e36:1 and e36:2, and in addition, lower amounts of e38:4, e40:8 and e40:9 of PC and PE.

### 3.3 Ether lipids of PE abundant in *O. ochengi* worms are also abundant in the nodule fluid of infected cattle

To unravel whether lipids abundant in *O. ochengi* accumulated in the host, bovine nodule fluid, which was collected from intradermal nodules of infected cattle, was analyzed for the presence of lipids potentially derived from *O. ochengi*. Phospholipids of bovine nodule fluid were mainly comprised of PC (76.9%), followed by PE (18.4%) and PI (3.9%) (Fig. 1A). PE and PI were lower in bovine nodule fluid than in *O. ochengi* worms. PS, which totaled ∼8% in *O. ochengi*, was barely detectable in nodule fluid. It is possible that the amount of PS was underestimated in nodule fluid because PS belongs to the acidic, less abundant phospholipids that are difficult to detect in complex matrices (Pettitt et al., 2006, Tuytten et al., 2006). Taken together, the phospholipid composition of bovine nodule fluid resembles that of *O. ochengi* worms, reflecting high levels of PE and PI. The most abundant molecular species of PC in bovine nodule fluid were 34:1, 34:2 36:1, 36:2, and 36:3 (Fig. 2A). Ether lipids of PC totaled 2.9% (e36:2) and 2.4% (e36:1), respectively, and similar amounts of e40:9 PC and e40:8 PC were also found. The PE molecular species distribution in nodule fluid were similar to that of *O. ochengi* worms. The ether lipids e36:2 PE and e36:1 PE constituted 23.3% and 17.0% in nodule fluid, respectively, and additional ether lipids (e36:3, e38:4, e40:8, e40:9) were also detected (Fig. 2B). We also measured the molecular species composition of PI, which amounts to 8.5% and 12.4% in female and male *O. ochengi* worms, respectively, and 3.9% in nodule fluid (Fig. 1A). The molecular species distribution of PI in nodule fluid was similar to that of the worms; i.e. dominated by 38:4 PI and 36:2 PI (Supplementary Fig. S1).

### 3.4 Phospholipids in uninfected and infected plasma or serum

Measurement of phospholipids in bovine nodules suggested that specific molecular species of PC, PE or PI, abundant in the *O. ochengi* worms, accumulated in the nodule fluid. Bovine serum phospholipids were analyzed to address the questions of whether the specific phospholipid molecular species in nodule fluid could be derived from the worm or from the host, and whether these lipids could also be detected in the serum. To this end, serum was obtained from uninfected and infected cattle and lipids extracted and measured by mass spectrometry. Bovine serum contained a very high amount of PC (97.5%), and low amounts of PE (0.5%) and PI (2.0%) (Fig. 1A). Other lipids (PG, PS, PA) were barely detectable. Interestingly, the PC content in nodule fluid (76.9%) was lower compared with bovine serum (97.5%), but not as low as in *O. ochengi* (57–58%), while the amounts of PE and PI (high in *O. ochengi*: 17–25% and 8–12%, respectively) were much higher in nodule fluid (17.4% and 3.9%, respectively) than in serum (0.5% and 2.0%, respectively). These results are in agreement with the scenario that PE and PI were released from *O. ochengi* into the nodule fluid, while PC in the nodule fluid was mostly derived from the host.

This finding is corroborated by the result that the molecular species distribution of PC did not greatly differ between bovine serum and nodule fluid. The major PC forms in bovine serum were 34:1, 34:2, 36:1, 36:2 and 36:3 (Fig. 2A), similar to the PC composition in nodule fluid. The ether lipids e36:2 PC and e36:1 PC totaled just 2.2% and 2.5% in serum, similar to nodule fluid. Therefore, PC molecular species in nodule fluid are mostly derived from bovine serum, where PC is extremely abundant.

In contrast, the molecular species distribution of PE was very different between bovine serum and nodule fluid (Fig. 2B). The ether lipids e36:2 PE and e36:1 PE constituted only 2.5% and 1.7%, respectively, in bovine serum, but were much more abundant in nodule fluid, similar to the amounts in *O. ochengi*. These results strongly suggest that a large proportion of ether PE in the nodule fluid is derived from *O. ochengi*. Further ether lipids of PE, e38:4, e40:8 and e40:9, which were abundant in worms and in nodule fluid, were barely detectable in serum. Therefore, the PE ether lipids in the nodule fluid were most likely derived from the worm.

A suitable biomarker in serum is expected to show low abundance in uninfected serum, but to increase after infection. Analysis of serum from uninfected and infected cattle revealed that e36:2 PE and e36:1 PE were present in low amounts, but they were not higher with infection (Fig.2B). The amounts of the other *O. ochengi*-specific ether lipids of PE (e38:4, e40:8 and e40:9) remained below detection limits in uninfected and infected serum samples. Therefore, the amounts of the PE ether lipids potentially released from the worms into the nodule fluid were very low in bovine serum.

The amount of PI (which was abundant in *O. ochengi* with 8–12%) was higher in nodule fluid (3.93%) than in uninfected serum (2.0%) (Fig. 1A). 38:4 PI was also the most abundant PI form in bovine serum, similar to worms and nodule fluid, but 36:2 PI (which was elevated in *O. ochengi* and nodule fluid) was scarce in serum. However, 36:2 PI was not increased in infected versus uninfected serum. The ether lipid e40:9 PI was detectable in *O. ochengi* worms and nodule fluid, but its content in plasma was extremely low. Other ether lipids of PI were also of very low abundance (Supplementary Fig. S1).

Next, phospholipids were measured in human plasma, and compared with the lipid pattern of *O. volvulus* worms. Uninfected human plasma contained almost exclusively PC, amounting to 96.2% of total phospholipids (Fig. 1B). PE and PI were also detected, but amounted to only 1.3% and 2.5% of total phospholipids, respectively, whereas PS and PG were below the detection limit in uninfected and infected plasma samples. Detection limits can be influenced by the matrix of the sample, e.g., the concentration of other lipids (Annesley, 2003). Therefore, the high amounts of PC may have interfered with the detection of PS and PG in plasma.

The major PC molecular species in human plasma were 34:1, 34:2, 36:1, 36:2, 36:3, 36:4, 36:5, 38:4, 38:5 and 38:6 (Fig. 3A). Together, the PC ether lipids e36:1 and e36:2 represented only 0.4% and 0.2% in uninfected human plasma, but more than 10% of total PC in *O. volvulus*. The amounts of ether lipids of PC were not higher in infected plasma (Fig. 3A, black versus white bars). This is probably due to the fact that parasite-specific ether lipids were highly diluted and masked by PC in human plasma.

We also looked into the possibility that molecular species of PE might represent suitable biomarkers, because PE totaled 16.7% in *O. volvulus*, but only 1.3% in human plasma (Fig. 1B). Therefore, even low amounts of *O. volvulus*-specific PE molecular species accumulating in the host might be detectable above the host-derived background of PE. The ether lipids e36:2 PE, e36:1 PE were abundant in *O. volvulus* worms, and further ether lipids identified were e38:3 PE and e40:8 PE. The ether lipids of PE in human plasma amounted to only between 1% and 3% of total PE (Fig. 3B). The amounts of e36:2 PE, e36:1 PE and e40:8 in human plasma did not change with infection status, while the amount of e38:3 PE was slightly, albeit not significantly, increased. Overall, the amount of parasite-derived PE released into the plasma was very low.

In addition, plasma from uninfected and infected jirds was analyzed for comparison with *L. sigmodontis*. Lipids, 34:1, 34:2, 36:1, 36:2, 36:3 PC were highly abundant, while the ether lipids of PC, e36:1 PC, e36:2 PC, e38:2 PC, e40:9, e40:8 PC and e40:7 PC were below 1% each in jird plasma (uninfected or infected). Therefore, similar to bovine serum and human plasma, no worm-specific ether lipids of PC accumulated in the infected jird plasma. Based on the very high PC content in jird plasma, worm-specific PC forms are presumably not suitable as biomarkers. The molecular species pattern of PE was similar to that of PC. Ether lipids of PE that were detected in *L. sigmodontis* (e36:1, e36:2, e38:2, e40:9) were <1% or even below the detection limit in uninfected or infected jird plasma (Fig. 4). Furthermore, the amounts of ether lipids of PE were similar in the plasma of uninfected or infected jirds. This indicates that the accumulation of specific ether lipids of PE, PC or PI in the bloodstream of the jirds was very limited.

## 4 Discussion

In the present study, we used direct infusion nano-ESI Q-TOF mass spectrometry to generate a comprehensive overview of the phospholipid content and molecular species distribution in three filarial nematode species: *O. ochengi, O. volvulus* and *L. sigmodontis,* and in the plasma or serum of uninfected and infected hosts (cattle, human and jird). A large number of phospholipid molecular species was detected in the nematodes. The two closely related nematode species, *O. ochengi* and *O. volvulus*, have highly similar phospholipid profiles, supporting the concept of using the *O. ochengi* model for studies of onchocerciasis. We identified several phospholipid forms which were abundant in the parasites, but barely detectable in the respective hosts. These molecular species were further investigated as putative biomarker candidates by quantitative comparison of phospholipids in bovine nodule fluid and in plasma or serum from uninfected and infected bovine, human and jird hosts. During these experiments, we established phospholipid measurements employing as little as 5 µl of bovine nodule fluid by direct infusion nano-ESI Q-TOF mass spectrometry.

The results on phospholipid analysis of *O. ochengi* and *O. volvulus* worms demonstrate that the nematodes contain high amounts of PC compared with PE and PI, while PG and PS are barely detectable. These results are in accordance with a previous report on *O. gibsoni* (Maloney and Semprevivo, 1991). Furthermore, all three worms contained high amounts of characteristic molecular species of ether lipids of PE (e36:2 PE, e36:1 PE, e40:9 PE, e40:8 PE) that accumulate in bovine nodule fluid, but that are absent or in low abundance in the host serum or plasma. Ether phospholipids have previously been found in other nematodes like *Caenorhabditis elegans*, where they are present at much lower amounts (Satouchi et al., 1993). In theory, the ether lipids of PE in the filarial nematodes or in the bovine nodule fluid could also be derived from the bacterial symbiont, *Wolbachia*, or from host metabolism. While the phospholipid composition of *Wolbachia* remains unknown, it has been shown that most bacteria are devoid of ether phospholipids. In principle it is possible that ether phospholipids (e.g. ether PE) accumulating in the bovine nodule fluid are not derived from the worm, but produced by bovine cells in the vicinity of the nodule. While this scenario cannot fully be ruled out, it is less likely, given that the nodule fluid phospholipid pattern in general seems to represent a mixture of worm and bovine lipids, e.g. with decreased amounts of PC and increased levels of PE compared with bovine serum (Fig.1A). Furthermore, not only *O. ochengi*-specific ether PE lipids accumulate in the nodule fluid, but also acyl lipids of PE and PI presumably derived from the worm (36:1 PC, 36:2 PI, 40:3 PI, Fig. 2, Supplementary Fig. S1). Therefore, it is likely that the ether lipids of PE and other phospholipids are derived from the nematodes, not from *Wolbachia* bacteria or from the host.

**Fig. 5:**
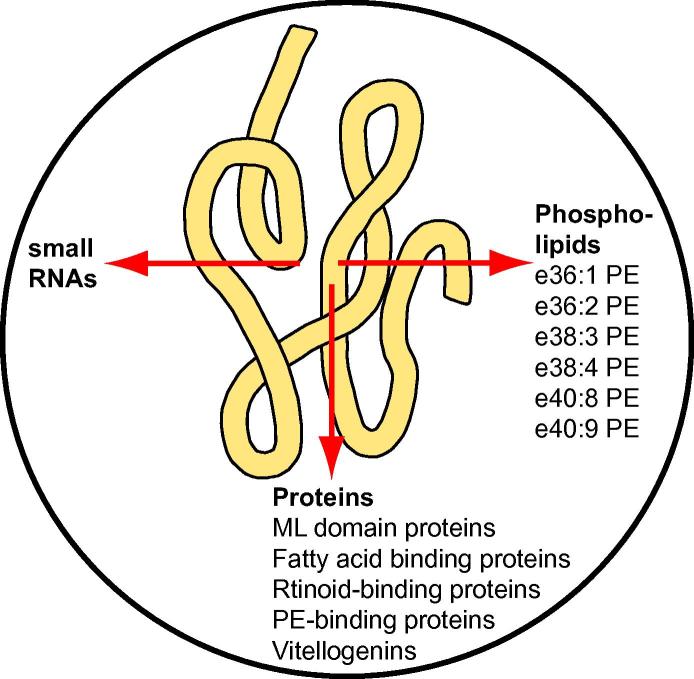
Release of nematode-specific phospholipids into nodule fluid. The fluid of intradermal nodules from cattle infected with *Onchocerca ochengi* can accumulate different nematode-derived compounds. In addition to small RNAs and proteins (Quintana et al., 2015, Armstrong et al., 2016), *O. ochengi*-specific ether (e) lipids of phosphatidylethanolamine (PE) were identified in the nodule fluid that can be used as biomarkers. The proteins identified in bovine nodule fluid encompass, among others, proteins predicted to be involved in lipid binding. Therefore, it is possible that the accumulation of nematode-derived phospholipids and lipid-binding proteins in nodule fluid is part of an active lipid transport process between the worms and the host. ML domain (MD2 domain lipid recognition) protein.

Thus, our results suggest that ether lipids of PE are released by the *O. ochengi* worm into the fluid of intradermal nodules (Fig. 5). Although not practicable for large scale screening programs, these molecular species can be employed as biomarkers in nodule fluid. In this scenario, lipids represent the third molecular class released by *O. ochengi* into nodule fluid, following previous analyses of small RNAs (Quintana et al., 2015) and proteins (Armstrong et al., 2016) (Fig. 5). Intriguingly, the adult secretome of filarial nematodes, including that of *O. ochengi* and *L. sigmodontis*, is rich in proteins that are predicted to bind to lipids, such as MD2 domain lipid recognition (ML domain) proteins, fatty acid and retinoid-binding proteins, PE-binding proteins, and vitellogenins (Armstrong et al., 2014, Armstrong et al., 2016). We speculate that these proteins could be involved in the transport of worm-derived lipids into host fluids, as well as capture of host-derived lipids for nutritional purposes.

While ether lipids of PE were clearly elevated in bovine nodule fluid where they can serve as nematode-specific biomarkers, these phospholipid molecular species were very low in infected bovine serum or human or jird plasma. The low abundance of these molecules in serum or plasma is probably due to high dilution effects. The distribution of PC, PE and PI in human plasma is comparable to previous reports, where contents of 70.8%, 3.4% and 4.4%, respectively, of total phospholipids, including sphingomyelin and lyso-phosphatidylcholine (LPC), were measured (Ismaiel et al., 2010). Thus, the very high content of PC in human plasma potentially masks the presence of nematode-specific PC molecular species, while PE molecular species are of much lower abundance. Nematode-specific ether lipids of PE were scarce in serum or plasma, and it was not possible to detect significant changes between uninfected and infected samples in the present study. However, it is possible that nematode-specific ether lipids, in particular those which were below the detection limit in serum or plasma (e.g., e38:4 PE, e40:9 PE, e40:8 PE; Fig. 4) could be identified as potential biomarkers after phospholipid enrichment via solid phase extraction, or via application of more sensitive mass spectrometry methods for phospholipid measurements, including triple quadrupole instruments or LC-MS.

Moreover, it is possible that the *Onchocerca*-specific lipids might accumulate in other tissues or fluids too, e.g., lymphatic fluid or urine. Using the *O. ochengi* infection, it would be intriguing to study whether the accumulation of these lipid molecular species in nodule fluid correlates with “fitness” of the parasite, e.g., after treatment with drugs that kill the adult worms. Other lipid classes such as triacylglycerol, sterols or sphingolipids (e.g., sphingomyeline, hexosylceramide, dihexosylceramide), which were detected in *O. ochengi* worms (results not presented) might also serve as potential biomarkers. Therefore, our results strongly suggest that phospholipids, in particular ether lipids of PE, are released from filarial nematodes into the host nodule fluid, but that more sensitive techniques are required to detect changes of these molecular phospholipid species in plasma or serum.
