# Supplementary material for: Lipid profiling of the filarial nematodes Onchocerca volvulus, Onchocerca ochengi and Litomosoides sigmodontis reveals the accumulation of nematode-specific ether phospholipids in the host
Source: Int J Parasitol. 2017 Dec;47(14):903–12. doi: 10.1016/j.ijpara.2017.06.001 (PMC5716430; doi:10.1016/j.ijpara.2017.06.001)
Supplement: Supplementary Table S1 [file mmc1.docx]

Supplementary Table S1. Phospholipids in individually pooled *Onchocerca volvulus* nematode samples. Lipid extracts from three different samples of *O. volvulus* were analyzed (sample 1 contained two males; sample 2, three females and two males; sample 3, three females and one male). The values represent individual measurements by direct infusion mass spectrometry averaged over 3-5 data points (Welti et al., 2002; Gasulla et al., 2013).

| Phospholipid class | Sample 1  (♂ = 2) | Sample 2  (♀ = 3, ♂ = 2) | Sample 3  (♀ = 3, ♂ = 1) |
| --- | --- | --- | --- |
|  | (mol% of total phospholipid) | | |
| PC | 69.4 | 59.9 | 57.8 |
| PE | 13.5 | 17.1 | 19.5 |
| PI | 1.7 | 0.9 | 0.8 |
| PG | 11.2 | 17.1 | 16.2 |
| PS | 4.2 | 5.1 | 5.8 |
|  |  |  |  |

PC, phosphatidylcholine; PE, phosphatidylethanolamine; PI, phosphatidylinositol; PG, phosphatidylglycerol; PS, phosphatidylserine.

**References**

Gasulla, F., vom Dorp, K., Dombrink, I., Zähringer, U., Gisch, N., Dormann, P., Bartels, D., 2013. The role of lipid metabolism in the acquisition of desiccation tolerance in *Craterostigma plantagineum*: A comparative approach. Plant J. 75, 726–741.

Welti, R., Li, W., Li, M., Sang, Y., Biesiada, H., Zhou, H.-E., Rajashekar, C.B., Williams, T.D., Wang, X., 2002. Profiling membrane lipids in plant stress responses. Role of phospholipase Da in freezing induced lipid changes in *Arabidopsis*. J. Biol. Chem. 277, 31994–32002.
