# Supplementary material for: Lipid profiling of the filarial nematodes Onchocerca volvulus, Onchocerca ochengi and Litomosoides sigmodontis reveals the accumulation of nematode-specific ether phospholipids in the host
Source: Int J Parasitol. 2017 Dec;47(14):903–12. doi: 10.1016/j.ijpara.2017.06.001 (PMC5716430; doi:10.1016/j.ijpara.2017.06.001)
Supplement: Supplementary Table S2 [file mmc2.docx]

Supplementary Table S2. Sum formulas and calculated m/z of phosphatidylethanolamine (PE) molecular species screened in worms and plasma. Proton adducts [M+H]^+^ were selected for MS/MS experiments during direct infusion nano electrospray ionization (ESI) -quadrupole time-of-flight (Q-TOF)-MS/MS analysis.

| Molecular Species | Sum Formula | Parental Ion (M+H)^+^  (m/z) | Sum Formula | Parental Ion (M+H)^+^  (m/z) | Neutral Loss (mass units) |
| --- | --- | --- | --- | --- | --- |
|  | PE (ester bond) | PE (ester bond) | ePE (ether bond) | ePE  (ether bond) |  |
| 28:0 (I.S.) | C_33_H_66_NO_8_P | 636.4599 | C_33_H_68_NO_7_P | 622.4806 | 141.0190 |
| 30:0 | C_35_H_70_NO_8_P | 664.4912 | C_35_H_72_NO_7_P | 650.5119 | 141.0190 |
| 30:1 | C_35_H_68_NO_8_P | 662.4755 | C_35_H_70_NO_7_P | 648.4963 | 141.0190 |
| 32:0 | C_37_H_74_NO_8_P | 692.5225 | C_37_H_76_NO_7_P | 678.5432 | 141.0190 |
| 32:1 | C_37_H_72_NO_8_P | 690.5068 | C_37_H_74_NO_7_P | 676.5276 | 141.0190 |
| 32:2 | C_37_H_70_NO_8_P | 688.4912 | C_37_H_72_NO_7_P | 674.5119 | 141.0190 |
| 32:3 | C_37_H_68_NO_8_P | 686.4755 | C_37_H_70_NO_7_P | 672.4963 | 141.0190 |
| 34:0 | C_39_H_78_NO_8_P | 720.5538 | C_39_H_80_NO_7_P | 706.5745 | 141.0190 |
| 34:1 | C_39_H_76_NO_8_P | 718.5381 | C_39_H_78_NO_7_P | 704.5589 | 141.0190 |
| 34:2 | C_39_H_74_NO_8_P | 716.5225 | C_39_H_76_NO_7_P | 702.5432 | 141.0190 |
| 34:3 | C_39_H_72_NO_8_P | 714.5068 | C_39_H_74_NO_7_P | 700.5276 | 141.0190 |
| 34:4 | C_39_H_70_NO_8_P | 712.4912 | C_39_H_72_NO_7_P | 698.5119 | 141.0190 |
| 36:0 | C_41_H_82_NO_8_P | 748.5851 | C_41_H_84_NO_7_P | 734.6058 | 141.0190 |
| 36:1 | C_41_H_80_NO_8_P | 746.5694 | C_41_H_82_NO_7_P | 732.5902 | 141.0190 |
| 36:2 | C_41_H_78_NO_8_P | 744.5538 | C_41_H_80_NO_7_P | 730.5745 | 141.0190 |
| 36:3 | C_41_H_76_NO_8_P | 742.5381 | C_41_H_78_NO_7_P | 728.5589 | 141.0190 |
| 36:4 | C_41_H_74_NO_8_P | 740.5225 | C_41_H_76_NO_7_P | 726.5432 | 141.0190 |
| 36:5 | C_41_H_72_NO_8_P | 738.5068 | C_41_H_74_NO_7_P | 724.5276 | 141.0190 |
| 36:6 | C_41_H_70_NO_8_P | 736.4912 | C_41_H_72_NO_7_P | 722.5119 | 141.0190 |
| 38:0 | C_43_H_86_NO_8_P | 776.6164 | C_43_H_88_NO_7_P | 762.6371 | 141.0190 |
| 38:1 | C_43_H_84_NO_8_P | 774.6007 | C_43_H_86_NO_7_P | 760.6215 | 141.0190 |
| 38:2 | C_43_H_82_NO_8_P | 772.5851 | C_43_H_84_NO_7_P | 758.6058 | 141.0190 |
| 38:3 | C_43_H_80_NO_8_P | 770.5694 | C_43_H_82_NO_7_P | 756.5902 | 141.0190 |
| 38:4 | C_43_H_78_NO_8_P | 768.5538 | C_43_H_80_NO_7_P | 754.5745 | 141.0190 |
| 38:5 | C_43_H_76_NO_8_P | 766.5381 | C_43_H_78_NO_7_P | 752.5589 | 141.0190 |
| 38:6 | C_43_H_74_NO_8_P | 764.5225 | C_43_H_76_NO_7_P | 750.5432 | 141.0190 |
| 38:7 | C_43_H_72_NO_8_P | 762.5068 | C_43_H_74_NO_7_P | 748.5276 | 141.0190 |
| 40:0 (I.S.) | C_45_H_90_NO_8_P | 804.6477 | C_45_H_92_NO_7_P | 790.6684 | 141.0190 |
| 40:1 | C_45_H_88_NO_8_P | 802.6320 | C_45_H_90_NO_7_P | 788.6528 | 141.0190 |
| 40:2 | C_45_H_86_NO_8_P | 800.6164 | C_45_H_88_NO_7_P | 786.6371 | 141.0190 |
| 40:3 | C_45_H_84_NO_8_P | 798.6007 | C_45_H_86_NO_7_P | 784.6215 | 141.0190 |
| 40:4 | C_45_H_82_NO_8_P | 796.5851 | C_45_H_84_NO_7_P | 782.6058 | 141.0190 |
| 40:5 | C_45_H_80_NO_8_P | 794.5694 | C_45_H_82_NO_7_P | 780.5902 | 141.0190 |
| 40:6 | C_45_H_78_NO_8_P | 792.5538 | C_45_H_80_NO_7_P | 778.5745 | 141.0190 |
| 40:7 | C_45_H_76_NO_8_P | 790.5381 | C_45_H_78_NO_7_P | 776.5589 | 141.0190 |
| 40:8 | C_45_H_74_NO_8_P | 788.5225 | C_45_H_76_NO_7_P | 774.5432 | 141.0190 |
| 40:9 | C_45_H_72_NO_8_P | 786.5068 | C_45_H_74_NO_7_P | 772.5276 | 141.0190 |
| 42:0 | C_47_H_94_NO_8_P | 832.6790 | C_47_H_96_NO_7_P | 818.6997 | 141.0190 |
| 42:1 | C_47_H_92_NO_8_P | 830.6633 | C_47_H_94_NO_7_P | 816.6841 | 141.0190 |
| 42:2 | C_47_H_90_NO_8_P | 828.6477 | C_47_H_92_NO_7_P | 814.6684 | 141.0190 |
| 42:3 | C_47_H_88_NO_8_P | 826.6320 | C_47_H_90_NO_7_P | 812.6528 | 141.0190 |
| 42:4 | C_47_H_86_NO_8_P | 824.6164 | C_47_H_88_NO_7_P | 810.6371 | 141.0190 |
| 42:5 | C_47_H_84_NO_8_P | 822.6007 | C_47_H_86_NO_7_P | 808.6215 | 141.0190 |
| 42:6 | C_47_H_82_NO_8_P | 820.5851 | C_47_H_84_NO_7_P | 806.6058 | 141.0190 |
| 42:7 | C_47_H_80_NO_8_P | 818.5694 | C_47_H_82_NO_7_P | 804.5902 | 141.0190 |
| 42:8 | C_47_H_78_NO_8_P | 816.5538 | C_47_H_80_NO_7_P | 802.5745 | 141.0190 |
| 42:9 | C_47_H_76_NO_8_P | 814.5381 | C_47_H_78_NO_7_P | 800.5589 | 141.0190 |
| 42:10 | C_47_H_74_NO_8_P | 812.5225 | C_47_H_76_NO_7_P | 798.5432 | 141.0190 |
| 44:0 | C_49_H_98_NO_8_P | 860.7103 | C_49_H_100_NO_7_P | 846.7310 | 141.0190 |
| 44:1 | C_49_H_96_NO_8_P | 858.6946 | C_49_H_98_NO_7_P | 844.7154 | 141.0190 |
| 44:2 | C_49_H_94_NO_8_P | 856.6790 | C_49_H_96_NO_7_P | 842.6997 | 141.0190 |
| 44:3 | C_49_H_92_NO_8_P | 854.6633 | C_49_H_94_NO_7_P | 840.6841 | 141.0190 |
| 44:4 | C_49_H_90_NO_8_P | 852.6477 | C_49_H_92_NO_7_P | 838.6684 | 141.0190 |
| 44:6 | C_49_H_86_NO_8_P | 848.6164 | C_49_H_88_NO_7_P | 834.6371 | 141.0190 |
| 44:7 | C_49_H_84_NO_8_P | 846.6007 | C_49_H_86_NO_7_P | 832.6215 | 141.0190 |
| 44:12 | C_49_H_74_NO_8_P | 836.5225 | C_49_H_76_NO_7_P | 822.5432 | 141.0190 |

I.S., internal standard; m/z, mass-to-charge ratio.
