# Supplementary material for: Lipid profiling of the filarial nematodes Onchocerca volvulus, Onchocerca ochengi and Litomosoides sigmodontis reveals the accumulation of nematode-specific ether phospholipids in the host
Source: Int J Parasitol. 2017 Dec;47(14):903–12. doi: 10.1016/j.ijpara.2017.06.001 (PMC5716430; doi:10.1016/j.ijpara.2017.06.001)
Supplement: Supplementary Table S3 [file mmc3.docx]

Supplementary Table S3. Sum formulas and calculated m/z of phosphatidylcholine (PC) molecular species screened in worms and plasma. Proton adducts [M+H]^+^ were selected for MS/MS experiments during direct infusion nano electrospray ionization (ESI) quadrupole-time-of-flight (Q-TOF) -MS/MS analysis.

| Molecular Species | Sum Formula | Parental Ion (M+H)^+^ (m/z) | Sum Formula | Parental Ion (M+H)^+^ (m/z) | Fragment Ion (mass units) |
| --- | --- | --- | --- | --- | --- |
|  | PC (ester bond) | PC (ester bond) | ePC (ether bond) | ePC  (ether bond) |  |
| 28:0 (I.S.) | C_36_H_72_NO_8_P | 678.5068 | C_36_H_74_NO_7_P | 664.5276 | 184.0739 |
| 30:0 | C_38_H_76_NO_8_P | 706.5381 | C_38_H_78_NO_7_P | 692.5589 | 184.0739 |
| 30:1 | C_38_H_74_NO_8_P | 704.5225 | C_38_H_76_NO_7_P | 690.5432 | 184.0739 |
| 32:0 | C_40_H_80_NO_8_P | 734.5694 | C_40_H_82_NO_7_P | 720.5902 | 184.0739 |
| 32:1 | C_40_H_78_NO_8_P | 732.5538 | C_40_H_80_NO_7_P | 718.5745 | 184.0739 |
| 32:2 | C_40_H_76_NO_8_P | 730.5381 | C_40_H_78_NO_7_P | 716.5589 | 184.0739 |
| 32:3 | C_40_H_74_NO_8_P | 728.5225 | C_40_H_76_NO_7_P | 714.5432 | 184.0739 |
| 34:0 | C_42_H_84_NO_8_P | 762.6007 | C_42_H_86_NO_7_P | 748.6215 | 184.0739 |
| 34:1 | C_42_H_82_NO_8_P | 760.5851 | C_42_H_84_NO_7_P | 746.6058 | 184.0739 |
| 34:2 | C_42_H_80_NO_8_P | 758.5694 | C_42_H_82_NO_7_P | 744.5902 | 184.0739 |
| 34:3 | C_42_H_78_NO_8_P | 756.5538 | C_42_H_80_NO_7_P | 742.5745 | 184.0739 |
| 34:4 | C_42_H_76_NO_8_P | 754.5381 | C_42_H_78_NO_7_P | 740.5589 | 184.0739 |
| 36:0 | C_44_H_88_NO_8_P | 790.6320 | C_44_H_90_NO_7_P | 776.6528 | 184.0739 |
| 36:1 | C_44_H_86_NO_8_P | 788.6164 | C_44_H_88_NO_7_P | 774.6371 | 184.0739 |
| 36:2 | C_44_H_84_NO_8_P | 786.6007 | C_44_H_86_NO_7_P | 772.6215 | 184.0739 |
| 36:3 | C_44_H_82_NO_8_P | 784.5851 | C_44_H_84_NO_7_P | 770.6058 | 184.0739 |
| 36:4 | C_44_H_80_NO_8_P | 782.5694 | C_44_H_82_NO_7_P | 768.5902 | 184.0739 |
| 36:5 | C_44_H_78_NO_8_P | 780.5538 | C_44_H_80_NO_7_P | 766.5745 | 184.0739 |
| 36:6 | C_44_H_76_NO_8_P | 778.5381 | C_44_H_78_NO_7_P | 764.5589 | 184.0739 |
| 38:0 | C_46_H_92_NO_8_P | 818.6633 | C_46_H_94_NO_7_P | 804.6841 | 184.0739 |
| 38:1 | C_46_H_90_NO_8_P | 816.6477 | C_46_H_92_NO_7_P | 802.6684 | 184.0739 |
| 38:2 | C_46_H_88_NO_8_P | 814.6320 | C_46_H_90_NO_7_P | 800.6528 | 184.0739 |
| 38:3 | C_46_H_86_NO_8_P | 812.6164 | C_46_H_88_NO_7_P | 798.6371 | 184.0739 |
| 38:4 | C_46_H_84_NO_8_P | 810.6007 | C_46_H_86_NO_7_P | 796.6215 | 184.0739 |
| 38:5 | C_46_H_82_NO_8_P | 808.5851 | C_46_H_84_NO_7_P | 794.6058 | 184.0739 |
| 38:6 | C_46_H_80_NO_8_P | 806.5694 | C_46_H_82_NO_7_P | 792.5902 | 184.0739 |
| 38:7 | C_46_H_78_NO_8_P | 804.5538 | C_46_H_80_NO_7_P | 790.5745 | 184.0739 |
| 40:0 (I.S.) | C_48_H_96_NO_8_P | 846.6946 | C_48_H_98_NO_7_P | 832.7154 | 184.0739 |
| 40:1 | C_48_H_94_NO_8_P | 844.6790 | C_48_H_96_NO_7_P | 830.6997 | 184.0739 |
| 40:2 | C_48_H_92_NO_8_P | 842.6633 | C_48_H_94_NO_7_P | 828.6841 | 184.0739 |
| 40:3 | C_48_H_90_NO_8_P | 840.6477 | C_48_H_92_NO_7_P | 826.6684 | 184.0739 |
| 40:4 | C_48_H_88_NO_8_P | 838.6320 | C_48_H_90_NO_7_P | 824.6528 | 184.0739 |
| 40:5 | C_48_H_86_NO_8_P | 836.6164 | C_48_H_88_NO_7_P | 822.6371 | 184.0739 |
| 40:6 | C_48_H_84_NO_8_P | 834.6007 | C_48_H_86_NO_7_P | 820.6215 | 184.0739 |
| 40:7 | C_48_H_82_NO_8_P | 832.5851 | C_48_H_84_NO_7_P | 818.6058 | 184.0739 |
| 40:8 | C_48_H_80_NO_8_P | 830.5694 | C_48_H_82_NO_7_P | 816.5902 | 184.0739 |
| 40:9 | C_48_H_78_NO_8_P | 828.5538 | C_48_H_80_NO_7_P | 814.5745 | 184.0739 |
| 42:0 | C_50_H_100_NO_8_P | 874.7259 | C_50_H_102_NO_7_P | 860.7467 | 184.0739 |
| 42:1 | C_50_H_98_NO_8_P | 872.7103 | C_50_H_100_NO_7_P | 858.7310 | 184.0739 |
| 42:2 | C_50_H_96_NO_8_P | 870.6946 | C_50_H_98_NO_7_P | 856.7154 | 184.0739 |
| 42:3 | C_50_H_94_NO_8_P | 868.6790 | C_50_H_96_NO_7_P | 854.6997 | 184.0739 |
| 42:4 | C_50_H_92_NO_8_P | 866.6633 | C_50_H_94_NO_7_P | 852.6841 | 184.0739 |
| 42:5 | C_50_H_90_NO_8_P | 864.6477 | C_50_H_92_NO_7_P | 850.6684 | 184.0739 |
| 42:6 | C_50_H_88_NO_8_P | 862.6320 | C_50_H_90_NO_7_P | 848.6528 | 184.0739 |
| 42:7 | C_50_H_86_NO_8_P | 860.6164 | C_50_H_88_NO_7_P | 846.6371 | 184.0739 |
| 42:8 | C_50_H_84_NO_8_P | 858.6007 | C_50_H_86_NO_7_P | 844.6215 | 184.0739 |
| 42:9 | C_50_H_82_NO_8_P | 856.5851 | C_50_H_84_NO_7_P | 842.6058 | 184.0739 |
| 42:10 | C_50_H_80_NO_8_P | 854.5694 | C_50_H_82_NO_7_P | 840.5902 | 184.0739 |
| 44:0 | C_52_H_104_NO_8_P | 902.7572 | C_52_H_106_NO_7_P | 888.7780 | 184.0739 |
| 44:1 | C_52_H_102_NO_8_P | 900.7416 | C_52_H_104_NO_7_P | 886.7623 | 184.0739 |
| 44:2 | C_52_H_100_NO_8_P | 898.7259 | C_52_H_102_NO_7_P | 884.7467 | 184.0739 |
| 44:3 | C_52_H_98_NO_8_P | 896.7103 | C_52_H_100_NO_7_P | 882.7310 | 184.0739 |
| 44:4 | C_52_H_96_NO_8_P | 894.6946 | C_52_H_98_NO_7_P | 880.7154 | 184.0739 |
| 44:6 | C_52_H_92_NO_8_P | 890.6633 | C_52_H_94_NO_7_P | 876.6841 | 184.0739 |
| 44:7 | C_52_H_90_NO_8_P | 888.6477 | C_52_H_92_NO_7_P | 874.6684 | 184.0739 |
| 44:12 | C_52_H_80_NO_8_P | 878.5694 | C_52_H_82_NO_7_P | 864.5902 | 184.0739 |

I.S., internal standard; m/z, mass-to-charge ratio.
