# Supplementary material for: Lipid profiling of the filarial nematodes Onchocerca volvulus, Onchocerca ochengi and Litomosoides sigmodontis reveals the accumulation of nematode-specific ether phospholipids in the host
Source: Int J Parasitol. 2017 Dec;47(14):903–12. doi: 10.1016/j.ijpara.2017.06.001 (PMC5716430; doi:10.1016/j.ijpara.2017.06.001)
Supplement: Supplementary Table S4 [file mmc4.docx]

Supplementary Table S4. Sum formulas and calculated m/z of phosphatidylglycerol (PG) molecular species screened in worms and plasma. Ammonium adducts [M+NH_4_]^+^ were selected for MS/MS experiments during direct infusion nano electrospray ionization (ESI) quadrupole-time-of-flight (Q-TOF) -MS/MS analysis.

| Molecular Species | Sum Formula | Parental Ion (M+NH_4_)^+^ (m/z) | Sum Formula | Parental Ion (M+NH_4_)^+^ (m/z) | Neutral Loss (mass units) |
| --- | --- | --- | --- | --- | --- |
| PG /ePG | PG (ester bond) | PG (ester bond) | ePG (ether bond) | PePG  (ether bond) |  |
| 28:0 (I.S.) | C_34_H_67_O_10_P | 684.4810 | C_34_H_69_O_9_P | 670.5018 | 189.0402 |
| 30:0 | C_36_H_71_O_10_P | 712.5123 | C_36_H_73_O_9_P | 698.5331 | 189.0402 |
| 30:1 | C_36_H_69_O_10_P | 710.4967 | C_36_H_71_O_9_P | 696.5174 | 189.0402 |
| 32:0 | C_38_H_75_O_10_P | 740.5436 | C_38_H_77_O_9_P | 726.5644 | 189.0402 |
| 32:1 | C_38_H_73_O_10_P | 738.5280 | C_38_H_75_O_9_P | 724.5487 | 189.0402 |
| 32:2 | C_38_H_71_O_10_P | 736.5123 | C_38_H_73_O_9_P | 722.5331 | 189.0402 |
| 32:3 | C_38_H_69_O_10_P | 734.4967 | C_38_H_71_O_9_P | 720.5174 | 189.0402 |
| 34:0 | C_40_H_79_O_10_P | 768.5749 | C_40_H_81_O_9_P | 754.5957 | 189.0402 |
| 34:1 | C_40_H_77_O_10_P | 766.5593 | C_40_H_79_O_9_P | 752.5800 | 189.0402 |
| 34:2 | C_40_H_75_O_10_P | 764.5436 | C_40_H_77_O_9_P | 750.5644 | 189.0402 |
| 34:3 | C_40_H_73_O_10_P | 762.5280 | C_40_H_75_O_9_P | 748.5487 | 189.0402 |
| 34:4 | C_40_H_71_O_10_P | 760.5123 | C_40_H_73_O_9_P | 746.5331 | 189.0402 |
| 36:0 | C_42_H_83_O_10_P | 796.6062 | C_42_H_85_O_9_P | 782.6270 | 189.0402 |
| 36:1 | C_42_H_81_O_10_P | 794.5906 | C_42_H_83_O_9_P | 780.6113 | 189.0402 |
| 36:2 | C_42_H_79_O_10_P | 792.5749 | C_42_H_81_O_9_P | 778.5957 | 189.0402 |
| 36:3 | C_42_H_77_O_10_P | 790.5593 | C_42_H_79_O_9_P | 776.5800 | 189.0402 |
| 36:4 | C_42_H_75_O_10_P | 788.5436 | C_42_H_77_O_9_P | 774.5644 | 189.0402 |
| 36:5 | C_42_H_73_O_10_P | 786.5280 | C_42_H_75_O_9_P | 772.5487 | 189.0402 |
| 36:6 | C_42_H_71_O_10_P | 784.5123 | C_42_H_73_O_9_P | 770.5331 | 189.0402 |
| 38:0 | C_44_H_87_O_10_P | 824.6375 | C_44_H_89_O_9_P | 810.6583 | 189.0402 |
| 38:1 | C_44_H_85_O_10_P | 822.6219 | C_44_H_87_O_9_P | 808.6426 | 189.0402 |
| 38:2 | C_44_H_83_O_10_P | 820.6062 | C_44_H_85_O_9_P | 806.6270 | 189.0402 |
| 38:3 | C_44_H_81_O_10_P | 818.5906 | C_44_H_83_O_9_P | 804.6113 | 189.0402 |
| 38:4 | C_44_H_79_O_10_P | 816.5749 | C_44_H_81_O_9_P | 802.5957 | 189.0402 |
| 38:5 | C_44_H_77_O_10_P | 814.5593 | C_44_H_79_O_9_P | 800.5800 | 189.0402 |
| 38:6 | C_44_H_75_O_10_P | 812.5436 | C_44_H_77_O_9_P | 798.5644 | 189.0402 |
| 38:7 | C_44_H_73_O_10_P | 810.5280 | C_44_H_75_O_9_P | 796.5487 | 189.0402 |
| 40:0 (I.S.) | C_46_H_91_O_10_P | 852.6688 | C_46_H_93_O_9_P | 838.6896 | 189.0402 |
| 40:1 | C_46_H_89_O_10_P | 850.6532 | C_46_H_91_O_9_P | 836.6739 | 189.0402 |
| 40:2 | C_46_H_87_O_10_P | 848.6375 | C_46_H_89_O_9_P | 834.6583 | 189.0402 |
| 40:3 | C_46_H_85_O_10_P | 846.6219 | C_46_H_87_O_9_P | 832.6426 | 189.0402 |
| 40:4 | C_46_H_83_O_10_P | 844.6062 | C_46_H_85_O_9_P | 830.6270 | 189.0402 |
| 40:5 | C_46_H_81_O_10_P | 842.5906 | C_46_H_83_O_9_P | 828.6113 | 189.0402 |
| 40:6 | C_46_H_79_O_10_P | 840.5749 | C_46_H_81_O_9_P | 826.5957 | 189.0402 |
| 40:7 | C_46_H_77_O_10_P | 838.5593 | C_46_H_79_O_9_P | 824.5800 | 189.0402 |
| 40:8 | C_46_H_75_O_10_P | 836.5436 | C_46_H_77_O_9_P | 822.5644 | 189.0402 |
| 40:9 | C_46_H_73_O_10_P | 834.5280 | C_46_H_75_O_9_P | 820.5487 | 189.0402 |
| 42:0 | C_48_H_95_O_10_P | 880.7001 | C_48_H_97_O_9_P | 866.7209 | 189.0402 |
| 42:1 | C_48_H_93_O_10_P | 878.6845 | C_48_H_95_O_9_P | 864.7052 | 189.0402 |
| 42:2 | C_48_H_91_O_10_P | 876.6688 | C_48_H_93_O_9_P | 862.6896 | 189.0402 |
| 42:3 | C_48_H_89_O_10_P | 874.6532 | C_48_H_91_O_9_P | 860.6739 | 189.0402 |
| 42:4 | C_48_H_87_O_10_P | 872.6375 | C_48_H_89_O_9_P | 858.6583 | 189.0402 |
| 42:5 | C_48_H_85_O_10_P | 870.6219 | C_48_H_87_O_9_P | 856.6426 | 189.0402 |
| 42:6 | C_48_H_83_O_10_P | 868.6062 | C_48_H_85_O_9_P | 854.6270 | 189.0402 |
| 42:7 | C_48_H_81_O_10_P | 866.5906 | C_48_H_83_O_9_P | 852.6113 | 189.0402 |
| 42:8 | C_48_H_79_O_10_P | 864.5749 | C_48_H_81_O_9_P | 850.5957 | 189.0402 |
| 42:9 | C_48_H_77_O_10_P | 862.5593 | C_48_H_79_O_9_P | 848.5800 | 189.0402 |
| 42:10 | C_48_H_75_O_10_P | 860.5436 | C_48_H_77_O_9_P | 846.5644 | 189.0402 |
| 44:0 | C_50_H_99_O_10_P | 908.7314 | C_50_H_101_O_9_P | 894.7522 | 189.0402 |
| 44:1 | C_50_H_97_O_10_P | 906.7158 | C_50_H_99_O_9_P | 892.7365 | 189.0402 |
| 44:2 | C_50_H_95_O_10_P | 904.7001 | C_50_H_97_O_9_P | 890.7209 | 189.0402 |
| 44:3 | C_50_H_93_O_10_P | 902.6845 | C_50_H_95_O_9_P | 888.7052 | 189.0402 |
| 44:4 | C_50_H_91_O_10_P | 900.6688 | C_50_H_93_O_9_P | 886.6896 | 189.0402 |
| 44:6 | C_50_H_87_O_10_P | 896.6375 | C_50_H_89_O_9_P | 882.6583 | 189.0402 |
| 44:7 | C_50_H_85_O_10_P | 894.6219 | C_50_H_87_O_9_P | 880.6426 | 189.0402 |
| 44:12 | C_50_H_75_O_10_P | 884.5436 | C_50_H_77_O_9_P | 870.5644 | 189.0402 |

I.S., internal standard; m/z, mass-to-charge ratio.
