# Supplementary material for: Lipid profiling of the filarial nematodes Onchocerca volvulus, Onchocerca ochengi and Litomosoides sigmodontis reveals the accumulation of nematode-specific ether phospholipids in the host
Source: Int J Parasitol. 2017 Dec;47(14):903–12. doi: 10.1016/j.ijpara.2017.06.001 (PMC5716430; doi:10.1016/j.ijpara.2017.06.001)
Supplement: Supplementary Table S5 [file mmc5.docx]

Supplementary Table S5. Sum formulas and calculated m/z of phosphatidylinositol (PI) molecular species screened in worms and plasma. Ammonium adducts [M+NH_4_]^+^ were selected for MS/MS experiments during direct infusion nano electrospray ionization (ESI) quadrupole-time-of-flight (Q-TOF) -MS/MS analysis.

| Molecular Species | Sum Formula | Parental Ion (M+NH_4_)^+^ (m/z) | Sum Formula | Parental Ion (M+NH_4_)^+^ (m/z) | Neutral Loss (mass units) |
| --- | --- | --- | --- | --- | --- |
|  | PI (ester bond) | PI (ester bond) | ePI (ether bond) | ePI  (ether bond) |  |
| 28:0 | C_37_H_71_O_13_P | 772.4971 | C_37_H_73_O_12_P | 758.5178 | 177.0563 |
| 30:0 | C_39_H_75_O_13_P | 800.5284 | C_39_H_77_O_12_P | 786.5491 | 177.0563 |
| 30:1 | C_39_H_73_O_13_P | 798.5127 | C_39_H_75_O_12_P | 784.5334 | 177.0563 |
| 32:0 | C_41_H_79_O_13_P | 828.5597 | C_41_H_81_O_12_P | 814.5804 | 177.0563 |
| 32:1 | C_41_H_77_O_13_P | 826.5440 | C_41_H_79_O_12_P | 812.5647 | 177.0563 |
| 32:2 | C_41_H_75_O_13_P | 824.5284 | C_41_H_77_O_12_P | 810.5491 | 177.0563 |
| 32:3 | C_41_H_73_O_13_P | 822.5127 | C_41_H_75_O_12_P | 808.5334 | 177.0563 |
| 34:0 (I.S.) | C_43_H_83_O_13_P | 856.5910 | C_43_H_85_O_12_P | 842.6117 | 177.0563 |
| 34:1 | C_43_H_81_O_13_P | 854.5753 | C_43_H_83_O_12_P | 840.5960 | 177.0563 |
| 34:2 | C_43_H_79_O_13_P | 852.5597 | C_43_H_81_O_12_P | 838.5804 | 177.0563 |
| 34:3 | C_43_H_77_O_13_P | 850.5440 | C_43_H_79_O_12_P | 836.5647 | 177.0563 |
| 34:4 | C_43_H_75_O_13_P | 848.5284 | C_43_H_77_O_12_P | 834.5491 | 177.0563 |
| 36:0 (I.S.) | C_45_H_87_O_13_P | 884.6223 | C_45_H_89_O_12_P | 870.6430 | 177.0563 |
| 36:1 | C_45_H_85_O_13_P | 882.6066 | C_45_H_87_O_12_P | 868.6273 | 177.0563 |
| 36:2 | C_45_H_83_O_13_P | 880.5910 | C_45_H_85_O_12_P | 866.6117 | 177.0563 |
| 36:3 | C_45_H_81_O_13_P | 878.5753 | C_45_H_83_O_12_P | 864.5960 | 177.0563 |
| 36:4 | C_45_H_79_O_13_P | 876.5597 | C_45_H_81_O_12_P | 862.5804 | 177.0563 |
| 36:5 | C_45_H_77_O_13_P | 874.5440 | C_45_H_79_O_12_P | 860.5647 | 177.0563 |
| 36:6 | C_45_H_75_O_13_P | 872.5284 | C_45_H_77_O_12_P | 858.5491 | 177.0563 |
| 38:0 | C_47_H_91_O_13_P | 912.6536 | C_47_H_93_O_12_P | 898.6743 | 177.0563 |
| 38:1 | C_47_H_89_O_13_P | 910.6379 | C_47_H_91_O_12_P | 896.6586 | 177.0563 |
| 38:2 | C_47_H_87_O_13_P | 908.6223 | C_47_H_89_O_12_P | 894.6430 | 177.0563 |
| 38:3 | C_47_H_85_O_13_P | 906.6066 | C_47_H_87_O_12_P | 892.6273 | 177.0563 |
| 38:4 | C_47_H_83_O_13_P | 904.5910 | C_47_H_85_O_12_P | 890.6117 | 177.0563 |
| 38:5 | C_47_H_81_O_13_P | 902.5753 | C_47_H_83_O_12_P | 888.5960 | 177.0563 |
| 38:6 | C_47_H_79_O_13_P | 900.5597 | C_47_H_81_O_12_P | 886.5804 | 177.0563 |
| 38:7 | C_47_H_77_O_13_P | 898.5440 | C_47_H_79_O_12_P | 884.5647 | 177.0563 |
| 40:0 | C_49_H_95_O_13_P | 940.6849 | C_49_H_97_O_12_P | 926.7056 | 177.0563 |
| 40:1 | C_49_H_93_O_13_P | 938.6692 | C_49_H_95_O_12_P | 924.6899 | 177.0563 |
| 40:2 | C_49_H_91_O_13_P | 936.6536 | C_49_H_93_O_12_P | 922.6743 | 177.0563 |
| 40:3 | C_49_H_89_O_13_P | 934.6379 | C_49_H_91_O_12_P | 920.6586 | 177.0563 |
| 40:4 | C_49_H_87_O_13_P | 932.6223 | C_49_H_89_O_12_P | 918.6430 | 177.0563 |
| 40:5 | C_49_H_85_O_13_P | 930.6066 | C_49_H_87_O_12_P | 916.6273 | 177.0563 |
| 40:6 | C_49_H_83_O_13_P | 928.5910 | C_49_H_85_O_12_P | 914.6117 | 177.0563 |
| 40:7 | C_49_H_81_O_13_P | 926.5753 | C_49_H_83_O_12_P | 912.5960 | 177.0563 |
| 40:8 | C_49_H_79_O_13_P | 924.5597 | C_49_H_81_O_12_P | 910.5804 | 177.0563 |
| 40:9 | C_49_H_77_O_13_P | 922.5440 | C_49_H_79_O_12_P | 908.5647 | 177.0563 |
| 42:0 | C_51_H_99_O_13_P | 968.7162 | C_51_H_101_O_12_P | 954.7369 | 177.0563 |
| 42:1 | C_51_H_97_O_13_P | 966.7005 | C_51_H_99_O_12_P | 952.7212 | 177.0563 |
| 42:2 | C_51_H_95_O_13_P | 964.6849 | C_51_H_97_O_12_P | 950.7056 | 177.0563 |
| 42:3 | C_51_H_93_O_13_P | 962.6692 | C_51_H_95_O_12_P | 948.6899 | 177.0563 |
| 42:4 | C_51_H_91_O_13_P | 960.6536 | C_51_H_93_O_12_P | 946.6743 | 177.0563 |
| 42:5 | C_51_H_89_O_13_P | 958.6379 | C_51_H_91_O_12_P | 944.6586 | 177.0563 |
| 42:6 | C_51_H_87_O_13_P | 956.6223 | C_51_H_89_O_12_P | 942.6430 | 177.0563 |
| 42:7 | C_51_H_85_O_13_P | 954.6066 | C_51_H_87_O_12_P | 940.6273 | 177.0563 |
| 42:8 | C_51_H_83_O_13_P | 952.5910 | C_51_H_85_O_12_P | 938.6117 | 177.0563 |
| 42:9 | C_51_H_81_O_13_P | 950.5753 | C_51_H_83_O_12_P | 936.5960 | 177.0563 |
| 42:10 | C_51_H_79_O_13_P | 948.5597 | C_51_H_81_O_12_P | 934.5804 | 177.0563 |
| 44:0 | C_53_H_103_O_13_P | 996.7475 | C_53_H_105_O_12_P | 982.7682 | 177.0563 |
| 44:1 | C_53_H_101_O_13_P | 994.7318 | C_53_H_103_O_12_P | 980.7525 | 177.0563 |
| 44:2 | C_53_H_99_O_13_P | 992.7162 | C_53_H_101_O_12_P | 978.7369 | 177.0563 |
| 44:3 | C_53_H_97_O_13_P | 990.7005 | C_53_H_99_O_12_P | 976.7212 | 177.0563 |
| 44:4 | C_53_H_95_O_13_P | 988.6849 | C_53_H_97_O_12_P | 974.7056 | 177.0563 |
| 44:6 | C_53_H_91_O_13_P | 984.6536 | C_53_H_93_O_12_P | 970.6743 | 177.0563 |
| 44:7 | C_53_H_89_O_13_P | 982.6379 | C_53_H_91_O_12_P | 968.6586 | 177.0563 |
| 44:12 | C_53_H_79_O_13_P | 972.5597 | C_53_H_81_O_12_P | 958.5804 | 177.0563 |

I.S., internal standard; m/z, mass-to-charge ratio.
