# Supplementary material for: Lipid profiling of the filarial nematodes Onchocerca volvulus, Onchocerca ochengi and Litomosoides sigmodontis reveals the accumulation of nematode-specific ether phospholipids in the host
Source: Int J Parasitol. 2017 Dec;47(14):903–12. doi: 10.1016/j.ijpara.2017.06.001 (PMC5716430; doi:10.1016/j.ijpara.2017.06.001)
Supplement: Supplementary Table S6 [file mmc6.docx]

Supplementary Table S6. Sum formulas and calculated m/z of phosphatidic acid (PA) molecular species screened in worms and plasma. Ammonium adducts [M+NH_4_]^+^ were selected for MS/MS experiments during direct infusion nano electrospray ionization (ESI) quadrupole-time-of-flight (Q-TOF) -MS/MS analysis.

| Molecular Species | Sum Formula | Parental Ion (M+NH_4_)^+^ (m/z) | Sum Formula | Parental Ion (M+NH_4_)^+^ (m/z) | Neutral Loss (mass units) |
| --- | --- | --- | --- | --- | --- |
|  | PA (ester bond) | PA (ester bond) | ePA (ether bond) | ePA  (ether bond) |  |
| 28:0 (I.S.) | C_31_H_61_O_8_P | 610.4442 | C_31_H_63_O_7_P | 596.465 | 115.0034 |
| 30:0 | C_33_H_65_O_8_P | 638.4755 | C_33_H_67_O_7_P | 624.4963 | 115.0034 |
| 30:1 | C_33_H_63_O_8_P | 636.4599 | C_33_H_65_O_7_P | 622.4806 | 115.0034 |
| 32:0 | C_35_H_69_O_8_P | 666.5068 | C_35_H_71_O_7_P | 652.5276 | 115.0034 |
| 32:1 | C_35_H_67_O_8_P | 664.4912 | C_35_H_69_O_7_P | 650.5119 | 115.0034 |
| 32:2 | C_35_H_65_O_8_P | 662.4755 | C_35_H_67_O_7_P | 648.4963 | 115.0034 |
| 32:3 | C_35_H_63_O_8_P | 660.4599 | C_35_H_65_O_7_P | 646.4806 | 115.0034 |
| 34:0 | C_37_H_73_O_8_P | 694.5381 | C_37_H_75_O_7_P | 680.5589 | 115.0034 |
| 34:1 | C_37_H_71_O_8_P | 692.5225 | C_37_H_73_O_7_P | 678.5432 | 115.0034 |
| 34:2 | C_37_H_69_O_8_P | 690.5068 | C_37_H_71_O_7_P | 676.5276 | 115.0034 |
| 34:3 | C_37_H_67_O_8_P | 688.4912 | C_37_H_69_O_7_P | 674.5119 | 115.0034 |
| 34:4 | C_37_H_65_O_8_P | 686.4755 | C_37_H_67_O_7_P | 672.4963 | 115.0034 |
| 36:0 | C_39_H_77_O_8_P | 722.5694 | C_39_H_79_O_7_P | 708.5902 | 115.0034 |
| 36:1 | C_39_H_75_O_8_P | 720.5538 | C_39_H_77_O_7_P | 706.5745 | 115.0034 |
| 36:2 | C_39_H_73_O_8_P | 718.5381 | C_39_H_75_O_7_P | 704.5589 | 115.0034 |
| 36:3 | C_39_H_71_O_8_P | 716.5225 | C_39_H_73_O_7_P | 702.5432 | 115.0034 |
| 36:4 | C_39_H_69_O_8_P | 714.5068 | C_39_H_71_O_7_P | 700.5276 | 115.0034 |
| 36:5 | C_39_H_67_O_8_P | 712.4912 | C_39_H_69_O_7_P | 698.5119 | 115.0034 |
| 36:6 | C_39_H_65_O_8_P | 710.4755 | C_39_H_67_O_7_P | 696.4963 | 115.0034 |
| 38:0 | C_41_H_81_O_8_P | 750.6007 | C_41_H_83_O_7_P | 736.6215 | 115.0034 |
| 38:1 | C_41_H_79_O_8_P | 748.5851 | C_41_H_81_O_7_P | 734.6058 | 115.0034 |
| 38:2 | C_41_H_77_O_8_P | 746.5694 | C_41_H_79_O_7_P | 732.5902 | 115.0034 |
| 38:3 | C_41_H_75_O_8_P | 744.5538 | C_41_H_77_O_7_P | 730.5745 | 115.0034 |
| 38:4 | C_41_H_73_O_8_P | 742.5381 | C_41_H_75_O_7_P | 728.5589 | 115.0034 |
| 38:5 | C_41_H_71_O_8_P | 740.5225 | C_41_H_73_O_7_P | 726.5432 | 115.0034 |
| 38:6 | C_41_H_69_O_8_P | 738.5068 | C_41_H_71_O_7_P | 724.5276 | 115.0034 |
| 38:7 | C_41_H_67_O_8_P | 736.4912 | C_41_H_69_O_7_P | 722.5119 | 115.0034 |
| 40:0 (I.S.) | C_43_H_85_O_8_P | 778.6320 | C_43_H_87_O_7_P | 764.6528 | 115.0034 |
| 40:1 | C_43_H_83_O_8_P | 776.6164 | C_43_H_85_O_7_P | 762.6371 | 115.0034 |
| 40:2 | C_43_H_81_O_8_P | 774.6007 | C_43_H_83_O_7_P | 760.6215 | 115.0034 |
| 40:3 | C_43_H_79_O_8_P | 772.5851 | C_43_H_81_O_7_P | 758.6058 | 115.0034 |
| 40:4 | C_43_H_77_O_8_P | 770.5694 | C_43_H_79_O_7_P | 756.5902 | 115.0034 |
| 40:5 | C_43_H_75_O_8_P | 768.5538 | C_43_H_77_O_7_P | 754.5745 | 115.0034 |
| 40:6 | C_43_H_73_O_8_P | 766.5381 | C_43_H_75_O_7_P | 752.5589 | 115.0034 |
| 40:7 | C_43_H_71_O_8_P | 764.5225 | C_43_H_73_O_7_P | 750.5432 | 115.0034 |
| 40:8 | C_43_H_69_O_8_P | 762.5068 | C_43_H_71_O_7_P | 748.5276 | 115.0034 |
| 40:9 | C_43_H_67_O_8_P | 760.4912 | C_43_H_69_O_7_P | 746.5119 | 115.0034 |
| 42:0 | C_45_H_89_O_8_P | 806.6633 | C_45_H_91_O_7_P | 792.6841 | 115.0034 |
| 42:1 | C_45_H_87_O_8_P | 804.6477 | C_45_H_89_O_7_P | 790.6684 | 115.0034 |
| 42:2 | C_45_H_85_O_8_P | 802.6320 | C_45_H_87_O_7_P | 788.6528 | 115.0034 |
| 42:3 | C_45_H_83_O_8_P | 800.6164 | C_45_H_85_O_7_P | 786.6371 | 115.0034 |
| 42:4 | C_45_H_81_O_8_P | 798.6007 | C_45_H_83_O_7_P | 784.6215 | 115.0034 |
| 42:5 | C_45_H_79_O_8_P | 796.5851 | C_45_H_81_O_7_P | 782.6058 | 115.0034 |
| 42:6 | C_45_H_77_O_8_P | 794.5694 | C_45_H_79_O_7_P | 780.5902 | 115.0034 |
| 42:7 | C_45_H_75_O_8_P | 792.5538 | C_45_H_77_O_7_P | 778.5745 | 115.0034 |
| 42:8 | C_45_H_73_O_8_P | 790.5381 | C_45_H_75_O_7_P | 776.5589 | 115.0034 |
| 42:9 | C_45_H_71_O_8_P | 788.5225 | C_45_H_73_O_7_P | 774.5432 | 115.0034 |
| 42:10 | C_45_H_69_O_8_P | 786.5068 | C_45_H_71_O_7_P | 772.5276 | 115.0034 |
| 44:0 | C_47_H_93_O_8_P | 834.6946 | C_47_H_95_O_7_P | 820.7154 | 115.0034 |
| 44:1 | C_47_H_91_O_8_P | 832.6790 | C_47_H_93_O_7_P | 818.6997 | 115.0034 |
| 44:2 | C_47_H_89_O_8_P | 830.6633 | C_47_H_91_O_7_P | 816.6841 | 115.0034 |
| 44:3 | C_47_H_87_O_8_P | 828.6477 | C_47_H_89_O_7_P | 814.6684 | 115.0034 |
| 44:4 | C_47_H_85_O_8_P | 826.6320 | C_47_H_87_O_7_P | 812.6528 | 115.0034 |
| 44:6 | C_47_H_81_O_8_P | 822.6007 | C_47_H_83_O_7_P | 808.6215 | 115.0034 |
| 44:7 | C_47_H_79_O_8_P | 820.5851 | C_47_H_81_O_7_P | 806.6058 | 115.0034 |
| 44:12 | C_47_H_69_O_8_P | 810.5068 | C_47_H_71_O_7_P | 796.5276 | 115.0034 |

I.S., internal standard; m/z, mass-to-charge ratio.
