# Supplementary material for: Lipid profiling of the filarial nematodes Onchocerca volvulus, Onchocerca ochengi and Litomosoides sigmodontis reveals the accumulation of nematode-specific ether phospholipids in the host
Source: Int J Parasitol. 2017 Dec;47(14):903–12. doi: 10.1016/j.ijpara.2017.06.001 (PMC5716430; doi:10.1016/j.ijpara.2017.06.001)
Supplement: Supplementary Table S7 [file mmc7.docx]

Supplementary Table S7. Sum formulas and calculated m/z of phosphatidylserine (PS) molecular species screened in worms and plasma. Proton adducts [M+H]^+^ were selected for MS/MS experiments during direct infusion nano electrospray ionization (ESI) quadrupole-time-of-flight (Q-TOF) analysis.

| Molecular Species | Sum Formula | Parental Ion (M+H)^+^ (m/z) | Sum Formula | Parental Ion (M+H)^+^ (m/z) | Neutral Loss (mass units) |
| --- | --- | --- | --- | --- | --- |
| PS /ePS | PS (ester bond) | PS (ester bond) | ePS (ether bond) | ePS  (ether bond) |  |
| 28:0 (I.S.) | C_34_H_66_NO_10_P | 680.4497 | C_34_H_68_NO_9_P | 666.4704 | 185.0089 |
| 30:0 | C_36_H_70_NO_10_P | 708.4810 | C_36_H_72_NO_9_P | 694.5017 | 185.0089 |
| 30:1 | C_36_H_68_NO_10_P | 706.4654 | C_36_H_70_NO_9_P | 692.4861 | 185.0089 |
| 32:0 | C_38_H_74_NO_10_P | 736.5123 | C_38_H_76_NO_9_P | 722.5330 | 185.0089 |
| 32:1 | C_38_H_72_NO_10_P | 734.4967 | C_38_H_74_NO_9_P | 720.5174 | 185.0089 |
| 32:2 | C_38_H_70_NO_10_P | 732.4810 | C_38_H_72_NO_9_P | 718.5017 | 185.0089 |
| 32:3 | C_38_H_68_NO_10_P | 730.4654 | C_38_H_70_NO_9_P | 716.4861 | 185.0089 |
| 34:0 | C_40_H_78_NO_10_P | 764.5436 | C_40_H_80_NO_9_P | 750.5643 | 185.0089 |
| 34:1 | C_40_H_76_NO_10_P | 762.5280 | C_40_H_78_NO_9_P | 748.5487 | 185.0089 |
| 34:2 | C_40_H_74_NO_10_P | 760.5123 | C_40_H_76_NO_9_P | 746.5330 | 185.0089 |
| 34:3 | C_40_H_72_NO_10_P | 758.4967 | C_40_H_74_NO_9_P | 744.5174 | 185.0089 |
| 34:4 | C_40_H_70_NO_10_P | 756.4810 | C_40_H_72_NO_9_P | 742.5017 | 185.0089 |
| 36:0 | C_42_H_82_NO_10_P | 792.5749 | C_42_H_84_NO_9_P | 778.5956 | 185.0089 |
| 36:1 | C_42_H_80_NO_10_P | 790.5593 | C_42_H_82_NO_9_P | 776.5800 | 185.0089 |
| 36:2 | C_42_H_78_NO_10_P | 788.5436 | C_42_H_80_NO_9_P | 774.5643 | 185.0089 |
| 36:3 | C_42_H_76_NO_10_P | 786.5280 | C_42_H_78_NO_9_P | 772.5487 | 185.0089 |
| 36:4 | C_42_H_74_NO_10_P | 784.5123 | C_42_H_76_NO_9_P | 770.5330 | 185.0089 |
| 36:5 | C_42_H_72_NO_10_P | 782.4967 | C_42_H_74_NO_9_P | 768.5174 | 185.0089 |
| 36:6 | C_42_H_70_NO_10_P | 780.4810 | C_42_H_72_NO_9_P | 766.5017 | 185.0089 |
| 38:0 | C_44_H_86_NO_10_P | 820.6062 | C_44_H_88_NO_9_P | 806.6269 | 185.0089 |
| 38:1 | C_44_H_84_NO_10_P | 818.5906 | C_44_H_86_NO_9_P | 804.6113 | 185.0089 |
| 38:2 | C_44_H_82_NO_10_P | 816.5749 | C_44_H_84_NO_9_P | 802.5956 | 185.0089 |
| 38:3 | C_44_H_80_NO_10_P | 814.5593 | C_44_H_82_NO_9_P | 800.5800 | 185.0089 |
| 38:4 | C_44_H_78_NO_10_P | 812.5436 | C_44_H_80_NO_9_P | 798.5643 | 185.0089 |
| 38:5 | C_44_H_76_NO_10_P | 810.5280 | C_44_H_78_NO_9_P | 796.5487 | 185.0089 |
| 38:6 | C_44_H_74_NO_10_P | 808.5123 | C_44_H_76_NO_9_P | 794.5330 | 185.0089 |
| 38:7 | C_44_H_72_NO_10_P | 806.4967 | C_44_H_74_NO_9_P | 792.5174 | 185.0089 |
| 40:0 (I.S.) | C_46_H_90_NO_10_P | 848.6375 | C_46_H_92_NO_9_P | 834.6582 | 185.0089 |
| 40:1 | C_46_H_88_NO_10_P | 846.6219 | C_46_H_90_NO_9_P | 832.6426 | 185.0089 |
| 40:2 | C_46_H_86_NO_10_P | 844.6062 | C_46_H_88_NO_9_P | 830.6269 | 185.0089 |
| 40:3 | C_46_H_84_NO_10_P | 842.5906 | C_46_H_86_NO_9_P | 828.6113 | 185.0089 |
| 40:4 | C_46_H_82_NO_10_P | 840.5749 | C_46_H_84_NO_9_P | 826.5956 | 185.0089 |
| 40:5 | C_46_H_80_NO_10_P | 838.5593 | C_46_H_82_NO_9_P | 824.5800 | 185.0089 |
| 40:6 | C_46_H_78_NO_10_P | 836.5436 | C_46_H_80_NO_9_P | 822.5643 | 185.0089 |
| 40:7 | C_46_H_76_NO_10_P | 834.5280 | C_46_H_78_NO_9_P | 820.5487 | 185.0089 |
| 40:8 | C_46_H_74_NO_10_P | 832.5123 | C_46_H_76_NO_9_P | 818.5330 | 185.0089 |
| 40:9 | C_46_H_72_NO_10_P | 830.4967 | C_46_H_74_NO_9_P | 816.5174 | 185.0089 |
| 42:0 | C_48_H_94_NO_10_P | 876.6688 | C_48_H_96_NO_9_P | 862.6895 | 185.0089 |
| 42:1 | C_48_H_92_NO_10_P | 874.6532 | C_48_H_94_NO_9_P | 860.6739 | 185.0089 |
| 42:2 | C_48_H_90_NO_10_P | 872.6375 | C_48_H_92_NO_9_P | 858.6582 | 185.0089 |
| 42:3 | C_48_H_88_NO_10_P | 870.6219 | C_48_H_90_NO_9_P | 856.6426 | 185.0089 |
| 42:4 | C_48_H_86_NO_10_P | 868.6062 | C_48_H_88_NO_9_P | 854.6269 | 185.0089 |
| 42:5 | C_48_H_84_NO_10_P | 866.5906 | C_48_H_86_NO_9_P | 852.6113 | 185.0089 |
| 42:6 | C_48_H_82_NO_10_P | 864.5749 | C_48_H_84_NO_9_P | 850.5956 | 185.0089 |
| 42:7 | C_48_H_80_NO_10_P | 862.5593 | C_48_H_82_NO_9_P | 848.5800 | 185.0089 |
| 42:8 | C_48_H_78_NO_10_P | 860.5436 | C_48_H_80_NO_9_P | 846.5643 | 185.0089 |
| 42:9 | C_48_H_76_NO_10_P | 858.5280 | C_48_H_78_NO_9_P | 844.5487 | 185.0089 |
| 42:10 | C_48_H_74_NO_10_P | 856.5123 | C_48_H_76_NO_9_P | 842.5330 | 185.0089 |
| 44:0 | C_50_H_98_NO_10_P | 904.7001 | C_50_H_100_NO_9_P | 890.7208 | 185.0089 |
| 44:1 | C_50_H_96_NO_10_P | 902.6845 | C_50_H_98_NO_9_P | 888.7052 | 185.0089 |
| 44:2 | C_50_H_94_NO_10_P | 900.6688 | C_50_H_96_NO_9_P | 886.6895 | 185.0089 |
| 44:3 | C_50_H_92_NO_10_P | 898.6532 | C_50_H_94_NO_9_P | 884.6739 | 185.0089 |
| 44:4 | C_50_H_90_NO_10_P | 896.6375 | C_50_H_92_NO_9_P | 882.6582 | 185.0089 |
| 44:6 | C_50_H_86_NO_10_P | 892.6062 | C_50_H_88_NO_9_P | 878.6269 | 185.0089 |
| 44:7 | C_50_H_84_NO_10_P | 890.5906 | C_50_H_86_NO_9_P | 876.6113 | 185.0089 |
| 44:12 | C_50_H_74_NO_10_P | 880.5123 | C_50_H_76_NO_9_P | 866.5330 | 185.0089 |

I.S.: internal standard, m/z : mass-to-charge ratio.
